# Can computerized clinical decision support systems improve practitioners' diagnostic test ordering behavior? A decision-maker-researcher partnership systematic review

**DOI:** 10.1186/1748-5908-6-88

**Published:** 2011-08-03

**Authors:** Pavel S Roshanov, John J You, Jasmine Dhaliwal, David Koff, Jean A Mackay, Lorraine Weise-Kelly, Tamara Navarro, Nancy L Wilczynski, R Brian Haynes

**Affiliations:** 1 Health Research Methodology Program, McMaster University, 1280 Main Street West, Hamilton, ON, Canada; 2 Department of Medicine, McMaster University, 1280 Main Street West, Hamilton, ON, Canada; 3 Hamilton Health Sciences, 1200 Main Street West, Hamilton, ON, Canada; 4 Department of Pediatrics, University of Alberta, 11402 University Avenue, Edmonton, AB, Canada; 5 Department of Radiology, McMaster University, 1280 Main Street West, Hamilton, ON, Canada; 6 Health Information Research Unit, Department of Clinical Epidemiology and Biostatistics, McMaster University, 1280 Main Street West, Hamilton, ON, Canada

## Abstract

**Background:**

Underuse and overuse of diagnostic tests have important implications for health outcomes and costs. Decision support technology purports to optimize the use of diagnostic tests in clinical practice. The objective of this review was to assess whether computerized clinical decision support systems (CCDSSs) are effective at improving ordering of tests for diagnosis, monitoring of disease, or monitoring of treatment. The outcome of interest was effect on the diagnostic test-ordering behavior of practitioners.

**Methods:**

We conducted a decision-maker-researcher partnership systematic review. We searched MEDLINE, EMBASE, Ovid's EBM Reviews database, Inspec, and reference lists for eligible articles published up to January 2010. We included randomized controlled trials comparing the use of CCDSSs to usual practice or non-CCDSS controls in clinical care settings. Trials were eligible if at least one component of the CCDSS gave suggestions for ordering or performing a diagnostic procedure. We considered studies 'positive' if they showed a statistically significant improvement in at least 50% of test ordering outcomes.

**Results:**

Thirty-five studies were identified, with significantly higher methodological quality in those published after the year 2000 (*p *= 0.002). Thirty-three trials reported evaluable data on diagnostic test ordering, and 55% (18/33) of CCDSSs improved testing behavior overall, including 83% (5/6) for diagnosis, 63% (5/8) for treatment monitoring, 35% (6/17) for disease monitoring, and 100% (3/3) for other purposes. Four of the systems explicitly attempted to reduce test ordering rates and all succeeded. Factors of particular interest to decision makers include costs, user satisfaction, and impact on workflow but were rarely investigated or reported.

**Conclusions:**

Some CCDSSs can modify practitioner test-ordering behavior. To better inform development and implementation efforts, studies should describe in more detail potentially important factors such as system design, user interface, local context, implementation strategy, and evaluate impact on user satisfaction and workflow, costs, and unintended consequences.

## Background

Much of medical care hinges on performing the right test, on the right patient, at the right time. Apart from their financial cost, diagnostic tests have downstream implications on care and, ultimately, patient outcomes. Yet, studies suggest wide variation in diagnostic test ordering behavior for seemingly similar patients [1-4]. This variation may be due to overuse or underuse of tests and may reflect inaccurate interpretation of results, rapid advances in diagnostic technology, and challenges in estimating tests' performance characteristics [5-10]. Thus, developing effective strategies to optimize healthcare practitioners' diagnostic test ordering behavior has become a major concern [11].

A variety of methods have been considered, including educational messages, reminders, and computerized clinical decision support systems (CCDSSs) [2,12-14]. For example, Thomas *et al. *[15] programmed a laboratory information system to automatically produce reminder messages that discourage future inappropriate use for each of nine diagnostic tests. A systematic review of strategies to change test-ordering behavior concluded that most interventions assessed were effective [2]. However, this review was limited by the low quality of primary studies. More recently, Shojania *et al. *[16] quantified the magnitude of improvements in processes of care from computer reminders delivered to physicians for any clinical purpose. Pooling data across randomized trials, they found a modest 3.8% median improvement (interquartile range [IQR], 15.9%) in adherence to test ordering reminders.

CCDSSs match characteristics of individual patients to a computerized knowledge base and provide patient-specific recommendations. The Health Information Research Unit (HIRU) at McMaster University previously conducted a systematic review assessing the effects of CCDSSs on practitioner performance and patient outcomes in 1994 [17], updated it in 1998 [18], and most recently in 2005 [19]. However, these reviews have not focused specifically on the use of diagnostic tests.

In this current update, we had the opportunity to partner with local hospital administration, clinical staff, and representatives of our regional health authority, in anticipation of major institutional investments in health information technology. Many new studies have been published in this field since our previous work in 2005 [19] allowing us to focus on randomized controlled trials (RCTs), with their lessened risk of bias. To better address the information needs of our decision-making partners, we focused on six separate topics for review: diagnostic test ordering, primary preventive care, drug prescribing, acute medical care, chronic disease management, and therapeutic drug monitoring and dosing. In this paper, we determine if CCDSSs improve practitioners' diagnostic test ordering behavior.

## Methods

We previously published detailed methods for conducting this systematic review available at http://www.implementationscience.com/content/5/1/12[20]. These methods are briefly summarized here, along with details specific to this review of CCDSSs for diagnostic test ordering.

### Research question

Do CCDSSs improve practitioners' diagnostic test ordering behavior?

### Partnering with decision makers

The research team engaged key decision makers early in the project to guide its design and endorse its funding application. Direction for the overall review was provided by senior administrators at Hamilton Health Sciences (one of Canada's largest teaching hospitals) and our regional health authority. JY (Department of Medicine) and DK (Chair, Department of Radiology) provided specific guidance for the area of diagnostic test ordering by selecting from each study the outcomes relevant to diagnostic testing. HIRU research staff searched for and selected trials for inclusion, as well as extracted and synthesised pertinent data. All partners worked together through the review process to facilitate knowledge translation, that is, to define whether and how to transfer findings into clinical practice.

### Search strategy

We previously published the details of our search strategy [20]. Briefly, we examined citations retrieved from MEDLINE, EMBASE, Ovid's Evidence-Based Medicine Reviews, and Inspec bibliographic databases up to 6 January 2010, and hand-searched the reference lists of included articles and relevant systematic reviews.

### Study selection

In pairs, our reviewers independently evaluated each study's eligibility for inclusion, and a third observer resolved disagreements. We first included all RCTs that assessed a CCDSS's effect on healthcare processes in which the system was used by healthcare professionals and provided patient-specific assessments or recommendations. We then selected trials of systems that gave direct recommendations to order or not to order a diagnostic test, or presented testing options, and measured impact on diagnostic processes. Trials of systems that simply gave advice for interpreting test results were excluded (such as Poels *et al. *[21]), as were trials of diagnostic systems that only reasoned through patient characteristics to suggest a diagnosis without making test recommendations (such as Bogusevicius *et al. *[22]). Systems that provided only information, such as cost of testing [23] or past test results [24] without actionable recommendations or options were also excluded.

### Data extraction

Pairs of reviewers independently extracted data from all eligible trials, including a wide range of system design and implementation characteristics, study methods, setting, funding sources, patient/provider characteristics, and effects on care process and clinical outcomes, adverse effects, effects on workflow, costs, and practitioner satisfaction. Disagreements were resolved by a third reviewer or by consensus. We attempted to contact primary authors of all included trials to confirm extracted data and to provide missing data, receiving a response from 69% (24/35).

### Assessment of study quality

We assessed the methodological quality of eligible trials with a 10-point scale consisting of five potential sources of bias, including concealment of allocation, appropriate unit of allocation, appropriate adjustment for baseline differences, appropriate blinding of assessment, and adequate follow-up [20]. For each source of bias, a score of 0 indicated the highest potential for bias, whereas a score of 2 indicated the lowest, generating a range of scores from 0 (lowest study quality) to 10 (highest study quality). We used a 2-tailed Mann-Whitney U test to assess whether the quality of trials has improved with time, comparing methodologic scores between trials published before the year 2000 and those published later.

### Assessment of CCDSS intervention effects

In determining effectiveness, we focused exclusively on diagnostic testing measures and defined these broadly to include performing physical examinations (*e.g.*, eye and foot exams), blood pressure measurements, as well as ordering laboratory, imaging, and functional tests. Patient outcomes were excluded from this study because, in general, they are most directly affected by treatment action and could not be attributed solely to diagnostic testing advice, especially in systems that also recommended therapy. Impact on patient outcomes and other process outcomes was assessed in our other current reviews on primary preventive care, drug prescribing, acute medical care, chronic disease management, and therapeutic drug monitoring and dosing.

Whenever possible, we classified systems as serving at least one of three purposes: disease monitoring (*e.g.*, measuring HbA_1c _in diabetes), treatment monitoring (*e.g.*, measuring liver enzymes at time of statin prescription), and diagnosis (*e.g.*, laboratory tests to detect source of fever). We classified trials in each area depending on whether they gave recommendations for that purpose and measured the outcome of those recommendations. Trials of systems for monitoring of medications with narrow therapeutic indexes, such as insulin or warfarin, are the focus of a separate report on CCDSSs for toxic drug monitoring and dosing and are not discussed here.

We looked for the intended direction of impact: to increase or to decrease testing. We considered a system effective if it changed, in the intended direction, a pre-specified primary outcome measuring use of diagnostic tests (2-tailed *p *< 0.05). If multiple pre-specified primary outcomes were reported, we considered a change in ≥50% of outcomes to represent effectiveness. We considered primary those outcomes reported by the author as 'primary' or 'main,' or if no such statements could be found, we considered the outcome used for sample size calculations to be primary. In the absence of a relevant primary outcome, we looked for a change in ≥50% of multiple pre-specified secondary outcomes. If there were no relevant pre-specified outcomes, systems that changed ≥50% of reported diagnostic process outcomes were considered effective. We included studies with multiple CCDSS arms in the count of 'positive' studies if any of the CCDSS arms showed a benefit over the control arm. These criteria are more specific than those used in our previous review [19]; therefore, some studies included in our earlier review [19] were re-categorised with respect to their effectiveness in this review.

### Data synthesis and analysis

We summarized data using descriptive measures, including proportions, medians, and ranges. Denominators vary in some proportions because not all trials reported relevant information. We conducted our analyses using SPSS, version 15.0. Given study-level differences in participants, clinical settings, disease conditions, interventions, and outcomes measured, we did not attempt a meta-analysis.

A sensitivity analysis was conducted to assess the possibility of biased results in studies with a mismatch between the unit of allocation (*e.g.*, clinicians) and the unit of analysis (*e.g.*, individual patients without adjustment for clustering). Success rates comparing studies with matched and mismatched analyses were compared using chi-square for comparisons. No differences in reported success were found for diagnostic process outcomes (Pearson X^2 ^= 0.44, *p = *0.51). Accordingly, results have been reported without distinction for mismatch.

## Results

**Figure 1:**
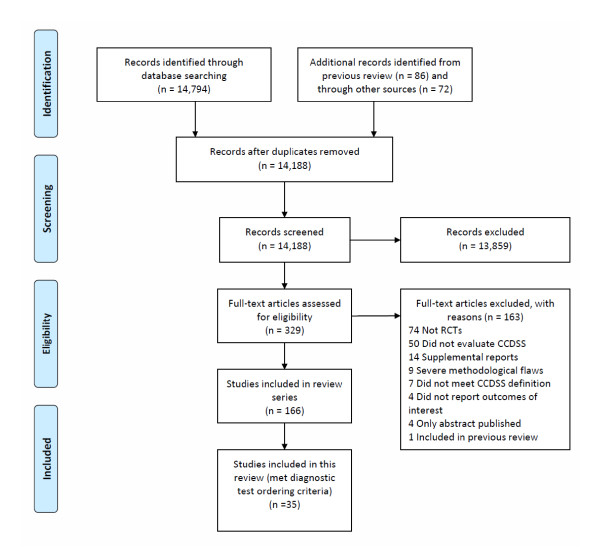
Flow diagram of included and excluded studies for the update 1 January 2004 to 6 January 2010 with specifics for diagnostic test ordering*. *Details provided in: Haynes RB *et al. *[20]. Two updating searches were performed, for 2004 to 2009 and to 6 January 2010 and the results of the search process are consolidated here.

Figure 1 shows the flow of included and excluded trials. Across all clinical indications, we identified 166 RCTs of CCDSSs and inter-reviewer agreement on study eligibility was high (unweighted Cohen's kappa, 0.93; 95% confidence interval [CI], 0.91 to 0.94). In this review, we included 35 trials described in 45 publications because they measured the impact on test ordering behavior of CCDSSs that gave suggestions for ordering or performing diagnostic tests [15,25-67]. Thirty-two included studies contributed outcomes to both this review and other CCDSS interventions in the series; four studies [34,37,41,68] to four reviews, 11 studies [25,32,33,35,36,39,40,42-44,46-49,51,57,61] to three reviews, and 17 studies [26-31,38,45,50,52-56,58-60,62-65] to two reviews; but we focused here only on diagnostic test ordering process outcomes.

Our assessment of trial quality is summarized in Additional file 1, Table S1; system characteristics in Additional file 2, Table S2; study characteristics in Additional file 3, Table S3; outcome data in Table 1 and Additional file 4, Table S4; and other CCDSS-related outcomes in Additional file 5, Table S5.

**Table 1:** Summary results for CCDSS trials of diagnostic test ordering^a^

Study	Methods score	Indication	No. of centres/providers/patients	Diagnostic process (DP) outcomes	CCDSS Effect^b^
**Disease Monitoring**					

Gilutz, 2009[25]	7	Reminders for monitoring and treatment of dyslipidemia in primary care patients with known coronary artery disease.	112*/600/7,448	Adequate frequency of lipoprotein monitoring.	**+**
Holbrook, 2009[26,27]	7	Web-based tracking of diabetes monitoring in adults in primary care.	18/46/511*	Semiannual measurement of glycated Hb, LDL-C, or albuminuria; semiannual foot surveillance; quarterly measurement of BP or BMI.	**+**
Maclean, 2009[28,29]	8	Reminders for the management of diabetes in primary care.	64*/132/7,412	Testing that was timely for A1C, lipids, serum creatinine, or urine microalbumin.	**+**
Peterson, 2008[30]	10	Visit reminders and patient-specific physician alerts and progress reports for organization of primary care in patients with type 2 diabetes.	24*/238/7,101	Improvement in Process of Care Index (annual BP monitoring; eye and foot exams; renal, HbA1c, and LDL-C testing).	**+**
Borbolla, 2007[31]	7	Recommendations for monitoring of BP in outpatients and primary care patients with chronic disease.	.../182*/2,315	BP measurement for appropriate patients.	**+**
Lester, 2006[32,33]	8	Recommendations for the management of dyslipidemia in primary care.	1/14/235*	Time to first measured LDL.-C.	**0**
Cobos^c^, 2005[34]	10	Recommendations for hypercholesterolemia therapy, follow-up visit frequency, and laboratory test ordering for patients with hypercholesterolemia in primary care.	42*/.../2,221	Number of patient assessments (lipids).	**0**
Plaza, 2005[35]	9	Recommendations for cost-effective management of asthma in primary care.	.../20*/198	Use of spirometry, conventional blood tests, total immunoglobulin E, skin allergy tests, or thorax radiography.	**0**
Sequist, 2005[36]	6	Reminders for management of diabetes and coronary artery disease in primary care.	20*/194/6,243	Receipt of annual cholesterol or dilated eye exams, or biennial HbA1c exams.	**0**
Tierney, 2005[37]	9	Recommendations for the management of asthma and chronic obstructive pulmonary disease in adults in primary care.	4/266*/706	Adherence to suggestions to obtain pulmonary function tests.	**0**
Mitchell 2004[38]	7	Feedback for identification, treatment, and control of hypertension in elderly patients in primary care.	52*/.../30,345	Patients with BP not measured.	**0 **
Eccles, 2002[39,40]	10	Recommendations for management of asthma or angina in adults in primary care.	62*/.../4,506	Adherence to angina guideline recommendations for recording/advising on BP; weight; electrocardiograms; thyroid function; Hb, lipid, cholesterol, blood glucose, and HbA1c levels; and assessment of lung function.	**0**
Demakis, 2000[41]	7	Reminders for screening, monitoring, and counselling in accordance with predefined standards of care in ambulatory care.	12*/275/12,989	Compliance with standards of care for coronary artery disease (lipid levels), hypertension (weight, exercise, sodium) and diabetes (glycosylated Hb, urinalysis, eye and foot exams).	**+**
Hetlevik, 1999[42-44]	8	Recommendations for diagnosis and management of hypertension, diabetes mellitus, and dyslipidemia in primary care.	56*/56/3,273	Hypertensive or diabetic patients without recorded data for BP, serum cholesterol, or BMI; and diabetic patients without HbA1c recorded.	**0**
Lobach, 1997[45]	6	Recommendations for the primary care of diabetes mellitus for outpatients, including screening, vaccination, and HbA1c monitoring.	1/58*/497	Compliance with diabetes management recommendations for foot, ophthalmologic, and complete physical exams; chronic glycemia monitoring; urine protein determination; and cholesterol levels.	**0**
Mazzuca, 1990[46]	7	Reminders for management of type 2 diabetes mellitus in outpatients.	4*/114/279	Adherence to recommendations for lab ordering for glycosylated Hb and fasting blood sugar; and initiation of home-monitored blood glucose.	**0**
Rogers, 1984[47-49]	4	Recommendations for the management of hypertension, obesity and renal disease in outpatients.	1/.../484*	Renal function or potassium exams, fundoscopy, or intravenous pyelograms for hypertensive patients; and renal function exams, urine analysis, or urine culture for patients with renal disease.	**0**

**Treatment Monitoring**					

Lo, 2009[50]	10	Alerts for ordering laboratory tests when prescribing new medications in primary care.	22*/366/2,765	Ordering appropriate baseline laboratory tests.	**0**
Matheny, 2008[51]	8	Reminders for routine medication laboratory monitoring in primary care.	20*/303/1922	Ordering appropriate laboratory tests.	**0**
Feldstein, 2006a[52,53]	10	Reminders to order laboratory tests when prescribing new medications in primary care.	15*/200/961	Completion of all baseline laboratory monitoring by day 25.	**+**
Palen, 2006[54]	9	Reminders for laboratory monitoring based on medication orders in primary care.	16/207*/26,586	Compliance with ordering the recommended laboratory tests.	**0**
Cobos^c^, 2005[34]	10	Recommendations for hypercholesterolemia therapy, follow-up visit frequency, and laboratory test ordering for patients with hypercholesterolemia in primary care.	42*/.../2,221	Number of patient assessments (aspartate or alanine aminotransferase, or creatine kinase).	**+**
Raebel, 2005[55]	8	Alerts to order laboratory tests when prescribing new medications in primary care.	.../.../400,000*	Drug dispensing with completed baseline laboratory monitoring.	**+**
McDonald, 1980[56]	5	Detection and management of mainly medication-related problems in outpatients.	1/31*/...	Adherence to reminders for recording a finding or ordering a test.	**+**
McDonald, 1976[57]	2	Recommendations for laboratory tests to detect potential medication-related events in adults attending a diabetes clinic.	1/.../226*	Compliance with ordering required tests for monitoring drug effects.	**+**
**Diagnosis**					

Sundaram, 2009[58]	7	Reminders for risk assessment and screening for human immunodeficiency virus in primary care.	5/32*/26,042	Change in human immunodeficiency virus testing rates.	**0**
Roukema, 2008[59]	7	Recommendations for the diagnostic management for children with fever without apparent source in the emergency department.	1/15/164*	Lab tests ordered.	**+**
Downs, 2006[60]	9	Prompts for the investigation and management of dementia in primary care.	35*/.../450	Detection of dementia and compliance with diagnostic guidelines.	**+**
Feldstein, 2006b[61]	8	Reminders for detection and treatment of osteoporosis in high-risk women in primary care who experienced a fracture.	15/159/311*	Receipt of bone mineral density measurement or osteoporosis medication.	**+**
Flottorp, 2002[62,63]	9	Recommendations for management of urinary tract infections in women or sore throat in primary care.	142*/.../...	Use of laboratory tests for sore throat or urinary tract infection.	**+**
McDonald, 1984[64]	6	Reminders for cancer screening (stool occult blood, mammogram), counselling (weight reduction), immunization (influenza, pneumococcal) in addition to >1000 physician behavior rules for outpatients.	1*/130/12467	Response to reminders for occult blood, cervical smear, hematocrit, chest roentgenogram, tuberculosis skin test, serum K, mammography, reticulocytes, iron/iron binding, liver enzymes, and tests for specific conditions.	**+**

**Other**					

Thomas, 2006[15]	8	Reminders to reduce inappropriate laboratory test orders in primary care.	85*/370/...	Targeted tests requested.	**+**
Javitt, 2005[65]	6	Recommendations for management of patients whose care deviates from recommended practices in primary care.	.../.../39,462*	Compliance with diagnostic test ordering recommendations.	**...**.
Bates, 1999[66]	8	Reminders to reduce redundant clinical laboratory tests in hospital inpatients.	1/.../16,586*	Tests performed after reminder triggered.	**+**
Overhage, 1997[68]	8	CCDSS identified 'corollary orders' (tests or treatments needed to monitor or ameliorate the effects of other tests or treatments) to prevent errors of omission for any of 87 target tests and treatments in hospital inpatients on a general medicine ward.	1*/92/2,181	Compliance with corollary orders.	**...**.
Tierney, 1988[67]	6	Provides information to reduce ordering of unnecessary diagnostic tests in primary care.	1/112/9,496*	Probability of abnormal study test.	**+**

Abbreviations: ACE, angiotensin-converting enzyme; BMI, body mass index; BP, blood pressure; CCDSS, computerized clinical decision support system; Hb, hemoglobin; LDL-C, low-density lipoprotein cholesterol.

*Unit of allocation.

^a^Ellipses (...) indicate item was not assessed or could not be evaluated.

^b^Outcomes are evaluated for effect as positive (+) or negative (-) for CCDSS, or no effect (0), based on the following hierarchy. An effect is defined as ≥50% of relevant outcomes showing a statistically significant difference (2*p*< 0.05):

1. If a single primary outcome is reported, *in which all components are applicable*, this is the only outcome evaluated.

2. If >1 primary outcome is reported, the ≥50% rule applies and only the primary outcomes are evaluated.

3. If no primary outcomes are reported (or only some of the primary outcome components are relevant) but overall analyses are provided, the overall analyses are evaluated as primary outcomes. Subgroup analyses are not considered.

4. If no primary outcomes or overall analyses are reported, or only some components of the primary outcome are relevant for the clinical care area, any reported prespecified outcomes are evaluated.

5. If no clearly pre-specified outcomes are reported, any available outcomes are considered.

6. If statistical comparisons are not reported, 'effect' is designated as not evaluated (...).

^c^Gives suggestions for monitoring of disease and treatment and is included in both categories. Outcomes were analyzed separately in each category but overall analysis of effectiveness (reported in text) was assessed for all diagnostic testing outcomes.

### Study quality

Details of our methodological quality assessment can be found in Additional file 1, Table S1. Fifty-four percent of trials concealed group allocation [26,27,30,32-35,37-40,42-44,50,52-55,60-63,66-68]; 51% allocated clusters (*e.g.*, entire clinics or wards) to minimize contamination between study groups [15,25,28-30,34,36,38-44,46,50-53,60,62-64,68]; 77% either showed no differences in baseline characteristics between study groups or adjusted accordingly [15,26-37,39,40,45-55,58,59,61-66,68]; 69% of trials achieved ≥90% follow-up for the appropriate unit of analysis [15,25,28-35,37,39-41,45,50-56,58-61,66,67]; and all but one used blinding or an objective outcome [45].

Most studies had good methodological quality (median quality score, 8; ranging from 2 to 10) and 63% (22/35) [15,25-38,50-55,58-61,65] were published after our previous search in September 2004. Study quality improved with time (median score before versus after year 2000, 7 versus 8, 2-tailed Mann-Whitney U = 44.5; *p *= 0.002), mainly because early trials did not conceal allocation and failed to achieve adequate follow-up.

### CCDSS and study characteristics

Systems' design and implementation characteristics are presented in Additional file 2, Table S2, but not all trials reported these details. CCDSSs in 80% of trials (28/35) gave advice at the time of care [25-27,30,31,34-37,39-51,54,56-64,66-68]; most were integrated with electronic medical records (82%; 27/33) [15,26,27,30-34,36,37,39,40,42-51,54,56-58,60-64,66-68] and some were integrated with computerized physician order entry (CPOE) systems (26%; 7/27) [31-33,37,50,54,67,68]; 77% (24/31) automatically obtained data needed to trigger recommendations from electronic medical records [15,26,27,30-34,36,37,39,40,45,46,50,51,54,56-58,60-64,66-68], while others relied on practitioners, existing non-prescribing staff, or research staff to enter data. In most trials (61%; 20/33) advice was delivered on a desktop or laptop computer [15,26,27,31,34,36-41,50,51,54,58-63,66-68], but other methods included personal digital assistants, email, or existing staff. Seventy-four percent (26/35) of systems were implemented in primary care [15,25-40,42-45,50-54,58,60-65,67]; 56% (14/25) were pilot tested [25,28-33,36,38,42-45,51,54,62,63,66,67]; and users of 59% (17/29) were trained [25-29,31-33,35,37,39-44,46,51,54,58-60,67]. Eighty-three percent of trials (29/35) declared that at least one author was involved in the development of the system [15,25-33,36,37,39-41,45-53,55-60,62-68]. In general, user interfaces were not described in detail. Additional file 3, Table S3 gives further description of the setting and method of CCDSS implementation.

The 35 trials included a total of 4,212 practitioners (median, 132; ranging from 14 to 600, when reported) caring for 626,382 patients (median, 2,765; ranging from 164 to 400,000, when reported) in 835 clinics (median, 15; ranging from 1 to 142, when reported) across 545 distinct sites (median, 4.5; ranging from 1 to 112, when reported).

Three trials did not declare a funding source [31,57,60]. Of those that did, 78% (25/32) were publically funded [15,25-30,36-38,41-51,54,56,58,59,61-64,66-68], 9% (3/32) received both private and public funding [39,40,55,61], and 13% (4/32) were conducted with private funds only [32-35,65].

### CCDSS effectiveness

Each system's impact on the use of diagnostic tests is summarized in Table 1, and Additional file 4, Table S4 provides a detailed description of test ordering outcomes. These outcomes were primary in 37% (13/35) of trials [15,25-27,31,41,50-53,55,58,61-63,66].

Fifty-six percent (18/33) of evaluated trials demonstrated an impact on the use of diagnostic tests [15,25-31,41,52,53,55-57,59-64,66,67] Two studies [65,68] met all eligibility criteria and included diagnostic process measures but were excluded from the assessment of effectiveness because they did not provide statistical comparisons of these measures.

### Disease monitoring

Systems in 49% (17/35) of trials (median quality score, 7; ranging from 4 to 10) gave recommendations for monitoring active conditions, all focusing on chronic diseases [25-49]. Their effectiveness for improving all processes of care and patient outcomes was assessed in our review on chronic disease management. Here we looked specifically for their impact on monitoring activity and found that 35% (6/17) increased appropriate monitoring [25-31,41].

In the context of diabetes, four of eight trials successfully increased timely monitoring of common targets such as HbA1c, blood lipids, blood pressure, urine albumin, and foot and eye health [26-30,41]. One of two systems that focused primarily on monitoring of hypertension was effective at increasing the frequency of appropriate blood pressure measurement [31]. One of three trials that focused on dyslipidemia improved monitoring of blood lipids [25]. Another three systems gave suggestions for monitoring of asthma [35,37,39,40], angina [39,40], chronic obstructive pulmonary disease (COPD) [37], and one for a combination of renal disease, obesity, and hypertension [47-49], but all failed to change testing behavior.

### Treatment monitoring

Systems in 23% of trials (8/35) [34,50-57] provided suggestions for laboratory monitoring of drug therapy. Trials in this area were generally recent and of high quality (median score, 8.5; range, 2 to 10; 75% (6/8) published since 2005). They targeted a wide range of medications (described in Additional file 4, Table S4) and are discussed in detail in our review of CCDSSs for drug prescribing, which looked for effects on prescribing behavior and patient outcomes. Focusing on their effectiveness for improving laboratory monitoring, we found that 63% (5/8) improved practices such as timely monitoring for adverse effects of medical therapy [34,52,53,55-57]. However, two of the trials demonstrating an impact were older and had low methodologic scores [56,57].

### Diagnosis

Systems in 17% of trials (6/35) [58-64] gave recommendations for ordering tests intended to aid diagnosis (median quality score, 7.5; ranging from 6 to 9) and 67% (4/6) were published since 2005 [58-61]. Eighty-three percent (5/6) successfully improved test ordering behavior [59-64]. Systems suggested tests to investigate suspected dementia in primary practice [60], to detect the source of fever for children in the emergency room [59], to increase bone mineral density measurements for diagnosing osteoporosis [61], to reduce unnecessary laboratory tests for diagnosing urinary tract infections or sore throats [62,63], to diagnose HIV [58], and to diagnose a host of conditions, including cancer, thyroid disorders, anemia, tuberculosis, and others [64].

### Other

Finally, five trials did not specify the clinical purpose of recommended tests [15,65-68], or suggested tests for several purposes but without data necessary to isolate the effects on testing for any one purpose. Three of five focused on reducing ordering rates and were successful [15,66,67]. Javitt *et al. *intended to increase test ordering and measured compliance with suggestions, but did not evaluate the outcome due to technical problems [65]. Overhage *et al. *meant to increase 'corollary orders' (tests to monitor the effects of other tests or treatments), but did not present statistical comparisons of their data on diagnostic process outcomes [68].

### Costs and practical process-related outcomes

Potentially important factors such as user satisfaction, adverse events, and impact on cost and workflow were rarely studied *(*see Additional file 5, Table S5*)*. Because most systems also gave recommendations for therapy, we were usually unable to isolate the effects of test-ordering suggestions on these factors, and here we discuss systems that gave only testing advice.

Two trials estimated statistically significant reductions in the cost of care, but estimates were small in one study [37] and imprecise (large confidence interval) in the other [28,29]. A third study estimated a relatively small reduction in annual laboratory costs ($35,000), but presented no statistical comparisons [66].

Three trials formally evaluated user satisfaction. One study found mixed satisfaction with a system for monitoring of diabetes and postulated that this was due to technical difficulties [26,27]. Another found that 78% of users felt CCDSS suggestion for ordering of HIV tests had an effect on their test-ordering practices, despite failing to show an effect of the CCDSS in the study [58]. The third study found that, regardless of high satisfaction with the local CPOE system, satisfaction with reminders about potentially redundant laboratory tests was lower (3.5 on a scale of 1 to 7) [66].

Only one study formally looked for adverse events caused by the CCDSS [66]. The system was designed to reduce potentially redundant clinical laboratory tests by giving reminders. Researchers assessed the potential for adverse events by checking for new abnormal test results for the same test performed after initial cancellation. Fifty-three percent of accepted reminders for a redundant test were followed by the same type of test within 72 hours, and 24% were abnormal, although only 4% provided new information and 1% led to changes in clinical management.

One study made a formal attempt to measure impact on user workflow and found that use of the CCDSS did not increase length of clinical encounters [45]. However, this outcome was not prespecified and the study may not have had adequate statistical power to detect an effect.

## Discussion

Our systematic review of RCTs of CCDSSs for diagnostic test ordering found that overall testing behavior was improved in just over one-half of trials. We considered studies 'positive' if they showed a statistically significant improvement in at least 50% of diagnostic process outcomes.

While the earliest RCT of a system for this purpose was published in 1976, most examples have appeared in the past five years, and evaluation methods have improved with time. Systems' diagnostic test ordering advice was most often intended to increase the ordering of certain tests in specific situations. Most systems suggested tests to diagnose new conditions, to monitor existing ones, or to monitor recently initiated drug treatments. Trials often demonstrated benefits in the areas of diagnosis and treatment monitoring, but were seldom effective for disease monitoring. All four systems that were explicitly meant to decrease unnecessary testing were successful [15,62,63,66,67]. CCDSSs may be better suited for some purposes than for others, but we need more trials and more detailed reporting of potential confounders, such as system design and implementation characteristics, to reliably assess the relationship between purpose and effectiveness.

Previous reviews have separately synthesized the literature on ways of improving diagnostic testing practice and on the effectiveness of CCDSSs [2,12-14,17-19,69]. Our current systematic review combines these areas and isolates the impact of CCDSS on diagnostic test ordering. However, several factors limited our analysis. Importantly, we chose not to evaluate effects on patient outcomes because many systems also gave treatment suggestions that affect these outcomes more directly than does test ordering advice. Some systems gave recommendations for testing but their respective studies did not measure the impact on test ordering practice and were, therefore, excluded from this review [70-72]. Only 37% of trials assessed impact on test ordering activity as a primary outcome, and others may not have had adequate statistical power to detect testing effects.

We did not determine the magnitude of effect in each study, there being no common metric for this, but simply considered studies 'positive' if they showed a statistically significant improvement in at least 50% of diagnostic process outcomes. As a result, some of the systems considered ineffective by our criteria reported statistically significant findings, but only for a minority of secondary or non-prespecified outcomes. Indeed, the limitations of this 'vote counting' [73] are well established and include increased risk of underestimating effect. However, our results remain essentially unchanged from our 2005 review [19] and are comparable to another major review [74], and a recent 'umbrella' review of high-quality systematic reviews of CCDSSs in hospital settings [75].

Vote counting prevented us from assessing publication bias but we believe that, along with selective outcome reporting, publication bias is a real issue in this literature because most systems were tested by their own developers.

We observed an improvement in trial quality over time, but this may simply reflect better reporting after standards such as Consolidated Standards of Reporting Trials (CONSORT) were widely adopted. Thirty-one percent of the authors we attempted to contact did not respond, and this may have particularly affected the quality of our extraction from older, less standardised reports.

While the number of RCTs has increased, the majority of these studies did not investigate or describe potentially important factors, including details of system design and implementation, costs and effects on user satisfaction, and workflow. Reporting such information is difficult under the space constraints of a trial publication, but supplementary reports may be an effective way to communicate these important details. One example comes from Flottorp *et al. *[62,63] who reported a process evaluation exploring the factors that affected the success of their CCDSS for management of sore throat and urinary tract infections. Feedback from practices showed that they were generally satisfied with installing and using the software, its technical performance, and with entering data. It also showed where they faced implementation challenges and which components of the intervention they used.

Our systematic review uncovered only three studies evaluating CCDSSs that give advice for the use of diagnostic imaging tests [35,61,64]. Effective decision support for ordering of imaging tests may be particularly relevant for the delivery of high quality, sustainable, modern healthcare, given the high cost and rapidly increasing use of such tests, and emerging concerns about cancer risk associated with exposure to medical radiation [11,76,77].

## Conclusions

Some CCDSSs improve practitioners' diagnostic test ordering behavior, but the determinants of success and failure remain unclear. CCDSSs may be better suited to improve testing for some purposes than others, but more trials and more detailed descriptions of system features and implementation are needed to evaluate this relationship reliably. Factors of interest to innovators who develop CCDSSs and decision makers considering local deployment are under-investigated or under-reported. To support the efforts of system developers, researchers should rigorously measure and report adverse effects of their system and impacts on user workflow and satisfaction, as well as details of their systems' design (*e.g.*, user interface characteristics and integration with other systems). To inform decision makers, researchers should report costs of design, development, and implementation.

## Competing interests

RBH, NLW, PSR, JJY, DK, JD, JAM, LWK, TN received support through the Canadian Institutes of Health Research Synthesis Grant: Knowledge Translation KRS 91791 for the submitted work. PSR was also supported by an Ontario Graduate Scholarship, a Canadian Institutes of Health Research Strategic Training Fellowship, and a Canadian Institutes of Health Research 'Banting and Best' Master's Scholarship. Additionally, PSR is a co-applicant for a patent concerning computerized decision support for anticoagulation, which was not discussed in this review, and has recently received awards from organizations that may benefit from the notion that information technology improves healthcare, including COACH (Canadian Organization for Advancement of Computers in Healthcare), the National Institutes of Health Informatics, and Agfa HealthCare Corp. JJY received funding to his institution through an Ontario Ministry of Health and Long-Term Care Career Scientist award; as well as funds paid to him for travel and accommodation for participation in a workshop sponsored by the Institute for Health Economics in Alberta, regarding optimal use of diagnostic imaging for low back pain. RBH is acquainted with several CCDSS developers and researchers, including authors of papers included in this review.

## Authors' contributions

RBH was responsible for study conception and design; acquisition, analysis and interpretation of data; critical revision of the manuscript; obtaining funding; and study supervision. He is the guarantor. PSR acquired, analyzed, and interpreted data; drafted and critically revised the manuscript; and provided statistical analysis. JJY acquired, analyzed, and interpreted data; and critically revised the manuscript. JD acquired data and drafted the manuscript. DK analyzed and interpreted data, and critically revised the manuscript. JAM acquired, analyzed, and interpreted data; drafted the manuscript; and provided statistical analysis as well as administrative, technical, or material support. LWK and TN acquired data and drafted the manuscript. NLW acquired, analyzed, and interpreted data; drafted the manuscript; and provided administrative, technical, or material support, as well as study supervision. All authors read and approved the final manuscript.

## Supplementary Material

Additional file 1 **Study methods scores for trials of diagnostic test ordering**. Methods scores for the included studies. Click here for file

Additional file 2 **CCDSS characteristics for trials of diagnostic test ordering**. CCDSS characteristics of the included studies. Click here for file

Additional file 3 **Study characteristics for trials of diagnostic test ordering**. Study characteristics of the included studies. Click here for file

Additional file 4 **Results for CCDSS trials of diagnostic test ordering**. Details results of the included studies. Click here for file

Additional file 5 **Costs and CCDSS process-related outcomes for trials of diagnostic test ordering**. Cost and CCDSS process-related outcomes for the included studies. Click here for file
