# Supplementary material for: Can computerized clinical decision support systems improve practitioners' diagnostic test ordering behavior? A decision-maker-researcher partnership systematic review
Source: Implement Sci. 2011 Aug 3;6:88. doi: 10.1186/1748-5908-6-88 (PMC3174115; doi:10.1186/1748-5908-6-88)
Supplement: Additional file 1 — Study methods scores for trials of diagnostic test ordering. Methods scores for the included studies. [file 1748-5908-6-88-S1.DOCX]

**Additional file 1, Table S1. Study methods scores for trials of diagnostic test ordering ^a^**

| **Study** | **Allocation concealed^b^** | **Cluster randomization** | **Adjustment for baseline differences** | **Objective outcome** | **Adequate follow-up** | **Total score** |
| --- | --- | --- | --- | --- | --- | --- |
| McDonald, 1976[57] | 0 | 0 | 0 | 2 | 0 | 2 |
| McDonald, 1980[56] | 0 | 1 | 0 | 2 | 2 | 5 |
| McDonald, 1984[64] | 0 | 2 | 2 | 2 | 0 | 6 |
| Rogers, 1984[47-49] | 0 | 0 | 2 | 2 | 0 | 4 |
| Tierney, 1988[67] | 2 | 0 | 0 | 2 | 2 | 6 |
| Mazzuca, 1990[46] | 0 | 2 | 2 | 2 | 1 | 7 |
| Lobach, 1997[45] | 0 | 1 | 2 | 1 | 2 | 6 |
| Overhage, 1997[68] | 2 | 2 | 2 | 2 | 0 | 8 |
| Bates, 1999[66] | 2 | 0 | 2 | 2 | 2 | 8 |
| Hetlevik, 1999[42-44] | 2 | 2 | 1 | 2 | 1 | 8 |
| Demakis, 2000[41] | 0 | 2 | 1 | 2 | 2 | 7 |
| Eccles, 2002[39, 40] | 2 | 2 | 2 | 2 | 2 | 10 |
| Flottorp, 2002[62, 63] | 2 | 2 | 2 | 2 | 1 | 9 |
| Mitchell, 2004[38] | 2 | 2 | 1 | 2 | 0 | 7 |
| Cobos, 2005[34] | 2 | 2 | 2 | 2 | 2 | 10 |
| Javitt, 2005[65] | 1 | 0 | 2 | 2 | 1 | 6 |
| Plaza, 2005[35] | 2 | 1 | 2 | 2 | 2 | 9 |
| Raebel, 2005[55] | 2 | 0 | 2 | 2 | 2 | 8 |
| Sequist, 2005[36] | 0 | 2 | 2 | 2 | 0 | 6 |
| Tierney, 2005[37] | 2 | 1 | 2 | 2 | 2 | 9 |
| Downs, 2006[60] | 2 | 2 | 1 | 2 | 2 | 9 |
| Feldstein, 2006a[52, 53] | 2 | 2 | 2 | 2 | 2 | 10 |
| Feldstein, 2006b[61] | 2 | 0 | 2 | 2 | 2 | 8 |
| Lester, 2006[32, 33] | 2 | 0 | 2 | 2 | 2 | 8 |
| Palen, 2006[54] | 2 | 1 | 2 | 2 | 2 | 9 |
| Thomas, 2006[15] | 0 | 2 | 2 | 2 | 2 | 8 |
| Borbolla, 2007[31] | 0 | 1 | 2 | 2 | 2 | 7 |
| Matheny, 2008[51] | 0 | 2 | 2 | 2 | 2 | 8 |
| Peterson, 2008[30] | 2 | 2 | 2 | 2 | 2 | 10 |
| Roukema, 2008[25] | 0 | 0 | 2 | 2 | 2 | 6 |
| Gilutz, 2009[39] | 0 | 2 | 1 | 2 | 2 | 7 |
| Holbrook, 2009[26, 27] | 2 | 0 | 2 | 2 | 1 | 7 |
| Lo, 2009[50] | 2 | 2 | 2 | 2 | 2 | 10 |
| Maclean, 2009[28, 29] | 0 | 2 | 2 | 2 | 2 | 8 |
| Sundaram, 2009[58] | 0 | 1 | 2 | 2 | 2 | 7 |

^a^Based on five individual items (score 2 = yes, 1 = partly, and 0 = no) and a summed total score (range 0 to 10). Because this review update included only randomized, controlled trials, the total score differs from that reported in the previous version of this review[19]: the item evaluating study type (randomized, quasi-randomized, or concurrent controls) has been replaced by one that evaluates use of concealed allocation (concealed, unclear, not concealed).

^b^If allocation concealment was not readily apparent from the description provided in the published article, the primary author of the trial confirmed or indicated that allocation was concealed.
