# Supplementary material for: Can computerized clinical decision support systems improve practitioners' diagnostic test ordering behavior? A decision-maker-researcher partnership systematic review
Source: Implement Sci. 2011 Aug 3;6:88. doi: 10.1186/1748-5908-6-88 (PMC3174115; doi:10.1186/1748-5908-6-88)
Supplement: Additional file 2 — CCDSS characteristics for trials of diagnostic test ordering. CCDSS characteristics of the included studies. [file 1748-5908-6-88-S2.DOCX]

**Additional file 2, Table S2. CCDSS characteristics for trials of diagnostic test ordering** ^a^

| **Study** | **Design** | | | **Interface description** | | | | | **Data entry source** | | | | | | **Methods for delivery of recommendations** | | | | | | | **CCDSS users** | | | | | | **Other characteristics** | | | | |
| --- | --- | --- | --- | --- | --- | --- | --- | --- | --- | --- | --- | --- | --- | --- | --- | --- | --- | --- | --- | --- | --- | --- | --- | --- | --- | --- | --- | --- | --- | --- | --- | --- |
|  | **Stand Alone** | **Integrated with EMR** | **Integrated with CPOE** | Graphic user interface | User must type | Drop down menus | Drag and drop | Other interface | **Automated through EMR** | **Project staff** | **Existing staff** | **Practitioner/decision-maker** | **Patient** | **Other data entry** | Desktop/Laptop computer | E-Mail | PDA | Pager | Project staff | Existing non-prescribing staff | Other Methods | **Trainees** | **Physicians** | **Advanced Practice Nurses** | **Physician Assistants** | **Pharmacists** | **Other health professionals** | Pilot tested | Users trained | Feedback at time of care | CCDSS suggested diagnoses/ treatments/procedures | Authors as developers |
| McDonald, 1976[57] | **-** | **+** | **-** | ? | ? | ? | ? | ? | **+** | **+** | **+** | **-** | **-** | **-** | - | - | - | - | - | + | - | **+** | **+** | **-** | **-** | **-** | **-** | - | - | + | + | + |
| McDonald, 1980[56] | **-** | **+** | **-** | ? | ? | ? | ? | ? | **+** | **-** | **-** | **-** | **-** | **-** | - | - | - | - | + | + | - | **+** | **+** | **+** | **-** | **-** | **-** | - | - | + | + | + |
| McDonald, 1984[64] | **-** | **+** | **?** | ? | ? | ? | ? | ? | **+** | **+** | **-** | **-** | **-** | **-** | - | - | - | - | - | + | - | **+** | **+** | **+** | **-** | **-** | **-** | - | - | + | + | + |
| Rogers, 1984[47-49] | **-** | **+** | **-** | ? | ? | ? | ? | ? | **?** | **?** | **?** | **?** | **?** | **?** | - | - | - | - | - | + | - | **-** | **+** | **-** | **-** | **-** | **-** | - | - | + | + | + |
| Tierney, 1988[67] | **-** | **+** | **+** | - | + | + | ? | ? | **+** | **-** | **-** | **+** | **-** | **-** | + | - | - | - | - | - | - | **+** | **+** | **-** | **-** | **-** | **-** | + | + | + | + | + |
| Mazzuca, 1990[46] | **-** | **+** | **?** | ? | ? | ? | ? | ? | **+** | **-** | **-** | **-** | **-** | **-** | - | - | - | - | - | + | - | **+** | **+** | **-** | **-** | **-** | **-** | - | + | + | + | + |
| Lobach, 1997[45] | **-** | **+** | **-** | + | ? | ? | ? | ? | **+** | **-** | **+** | **+** | **-** | **-** | - | - | - | - | - | + | - | **-** | **+** | **+** | **+** | **-** | **-** | + | - | + | + | + |
| Overhage, 1997[68] | **-** | **+** | **+** | ? | ? | ? | ? | ? | **+** | **-** | **-** | **+** | **-** | **-** | + | - | - | - | - | - | - | **+** | **+** | **-** | **-** | **-** | **-** | - | - | + | + | + |
| Bates, 1999[66] | **-** | **+** | **-** | - | + | - | - | - | **+** | **-** | **-** | **-** | **-** | **-** | + | - | - | - | - | - | - | **+** | **+** | **+** | **-** | **-** | **+** | + | - | + | ? | + |
| Hetlevik, 1999[42-44] | **-** | **+** | **-** | ? | ? | ? | ? | ? | **?** | **?** | **?** | **?** | **?** | **?** | ? | ? | ? | ? | ? | ? | ? | **-** | **+** | **-** | **+** | **-** | **-** | + | + | + | + | - |
| Demakis, 2000[41] | **+** | **-** | **-** | - | + | - | - | + | **-** | **-** | **-** | **+** | **-** | **+** | + | - | - | - | - | + | - | **+** | **+** | **-** | **-** | **-** | **-** | - | + | + | + | + |
| Eccles, 2002[39, 40] | **-** | **+** | **-** | ? | ? | ? | ? | ? | **+** | **-** | **-** | **+** | **-** | **-** | + | - | - | - | - | - | - | **-** | **+** | **-** | **-** | **-** | **-** | - | + | + | + | + |
| Flottorp, 2002[62, 63] | **-** | **+** | **?** | + | ? | ? | ? | + | **+** | **+** | **-** | **+** | **-** | **-** | + | - | - | - | - | - | - | **-** | **+** | **-** | **+** | **-** | **-** | + | - | + | + | + |
| Mitchell, 2004[38] | **-** | **+** | **-** | - | - | - | - | - | **-** | **-** | **-** | **-** | **-** | **+** | + | - | - | - | - | - | - | **-** | **+** | **-** | **-** | **-** | **-** | + | - | - | + | ? |
| Cobos, 2005[34] | **-** | **+** | **-** | ? | ? | ? | ? | ? | **+** | **-** | **-** | **-** | **-** | **-** | + | - | - | - | - | - | - | **-** | **+** | **-** | **-** | **-** | **-** | ? | ? | + | + | ? |
| Javitt, 2005[65] | **+** | **-** | **-** | ? | ? | ? | ? | ? | **-** | **-** | **-** | **-** | **-** | **+** | - | - | - | - | - | - | + | **-** | **+** | **-** | **-** | **-** | **-** | ? | ? | - | + | + |
| Plaza, 2005[35] | **+** | **-** | **-** | ? | ? | ? | ? | ? | **-** | **-** | **-** | **+** | **-** | **-** | - | - | + | - | - | - | - | **-** | **+** | **-** | **-** | **-** | **-** | ? | + | + | + | ? |
| Raebel, 2005[55] | **?** | **?** | **?** | ? | ? | ? | ? | ? | **?** | **?** | **?** | **?** | **?** | **?** | ? | ? | ? | ? | ? | ? | ? | **-** | **+** | **-** | **-** | **+** | **-** | ? | ? | - | + | + |
| Sequist, 2005[36] | **-** | **+** | **-** | + | + | + | ? | ? | **+** | **-** | **-** | **-** | **-** | **-** | + | - | - | - | - | - | + | **+** | **+** | **-** | **-** | **-** | **-** | + | - | + | + | + |
| Tierney, 2005[37] | **-** | **+** | **+** | + | + | ? | ? | + | **+** | **-** | **-** | **+** | **-** | **-** | + | - | - | - | - | - | + | **+** | **+** | **-** | **-** | **+** | **-** | ? | + | + | + | + |
| Downs, 2006[60] | **-** | **+** | **?** | ? | ? | ? | ? | ? | **+** | **-** | **-** | **-** | **-** | **-** | + | - | - | - | - | - | - | **-** | **+** | **+** | **-** | **-** | **-** | ? | + | + | + | + |
| Feldstein, 2006a[52, 53] | **?** | **?** | **?** | ? | ? | ? | ? | ? | **?** | **?** | **?** | **?** | **?** | **?** | - | - | - | - | - | - | + | **-** | **+** | **+** | **-** | **-** | **-** | ? | ? | - | + | + |
| Feldstein, 2006b[61] | **-** | **+** | **?** | + | - | ? | ? | - | **+** | **-** | **-** | **-** | **-** | **-** | + | + | - | - | - | - | - | **-** | **+** | **-** | **-** | **-** | **-** | ? | ? | + | + | ? |
| Lester, 2006[32, 33] | **-** | **+** | **+** | + | - | - | - | + | **+** | **-** | **-** | **-** | **-** | **-** | - | + | - | - | - | - | - | **-** | **+** | **-** | **-** | **-** | **-** | + | + | - | + | + |
| Palen, 2006[54] | **-** | **+** | **+** | ? | ? | ? | ? | ? | **+** | **-** | **-** | **-** | **-** | **-** | + | - | - | - | - | - | - | **-** | **+** | **-** | **-** | **-** | **-** | + | + | + | + | ? |
| Thomas, 2006[15] | **-** | **+** | **?** | ? | ? | ? | ? | ? | **+** | **-** | **-** | **-** | **-** | **-** | + | - | - | - | - | - | + | **-** | **+** | **-** | **-** | **-** | **-** | ? | ? | - | + | + |
| Borbolla, 2007[31] | **-** | **+** | **+** | ? | + | ? | ? | ? | **+** | **+** | **+** | **-** | **-** | **-** | + | - | - | - | - | - | - | **-** | **+** | **-** | **+** | **-** | **-** | + | + | + | + | + |
| Matheny, 2008[51] | **-** | **+** | **-** | + | - | - | - | + | **+** | **-** | **-** | **-** | **-** | **-** | + | - | - | - | - | - | - | **-** | **+** | **-** | **-** | **-** | **-** | + | + | + | + | + |
| Peterson, 2008[30] | **-** | **+** | **-** | ? | ? | ? | ? | ? | **+** | **+** | **-** | **-** | **-** | **+** | - | - | - | - | - | + | + | **-** | **+** | **-** | **+** | **-** | **+** | + | - | + | + | + |
| Roukema, 2008[59] | **+** | **-** | **-** | + | + | + | ? | ? | **-** | **-** | **+** | **-** | **-** | **-** | + | - | - | - | - | - | - | **-** | **+** | **+** | **-** | **-** | **-** | ? | + | + | + | + |
| Gilutz, 2009[25] | **+** | **-** | **-** | ? | ? | ? | ? | ? | **-** | **+** | **-** | **-** | **-** | **-** | - | - | - | - | - | - | + | **-** | **+** | **-** | **-** | **-** | **+** | + | + | + | + | + |
| Holbrook, 2009[26, 27] | **-** | **+** | **-** | + | - | ? | ? | ? | **+** | **-** | **-** | **+** | **-** | **-** | + | - | - | - | - | - | - | **-** | **+** | **+** | **-** | **-** | **-** | - | + | + | + | + |
| Lo, 2009[50] | **-** | **+** | **+** | + | - | + | ? | ? | **+** | **-** | **-** | **-** | **-** | **-** | + | - | - | - | - | - | - | **+** | **+** | **+** | **+** | **-** | **-** | - | - | + | + | + |
| Maclean, 2009[28, 29] | **+** | **-** | **-** | - | - | - | - | - | **-** | **+** | **-** | **-** | **-** | **-** | - | - | - | - | - | - | + | **-** | **+** | **+** | **+** | **-** | **-** | + | + | - | + | + |
| Sundaram, 2009[58] | **-** | **+** | **-** | + | ? | + | - | + | **+** | **-** | **-** | **-** | **-** | **-** | + | - | - | - | - | - | + | **-** | **+** | **+** | **-** | **-** | **-** | - | + | + | + | + |

Abbreviations: CCDSS, computerized clinical decision support system; CPOE, computerized physician order entry system; EMR, electronic medical record; PDA, personal digital assistant.

^a^Symbol key: +, characteristic present; -, characteristic absent; ~, characteristic sometimes present; ?, unstated or uncertain.
