# Supplementary material for: Can computerized clinical decision support systems improve practitioners' diagnostic test ordering behavior? A decision-maker-researcher partnership systematic review
Source: Implement Sci. 2011 Aug 3;6:88. doi: 10.1186/1748-5908-6-88 (PMC3174115; doi:10.1186/1748-5908-6-88)
Supplement: Additional file 3 — Study characteristics for trials of diagnostic test ordering. Study characteristics of the included studies. [file 1748-5908-6-88-S3.DOCX]

**Additional file 3, Table S3. Study characteristics for trials of diagnostic test ordering^a^**

| **Study (country)** | **Methods score^b^** | **Funding source** | **Indication** | **No. of practitioners / patients** | **Setting^c^(No. of clinics / sites)** | **CCDSS intervention** | **Comparison** |
| --- | --- | --- | --- | --- | --- | --- | --- |
| **Disease Monitoring** | | | | | | | |
| Gilutz, 2009[25], Israel | 7 | Public | Lipid monitoring and treatment of patients previously hospitalised with coronary artery disease (CAD) and followed up in primary care. | 600 / 7448 | •Primary care •Community-based clinic (112/112) | CCDSS collected data from 3 databases (discharge and diagnosis; laboratory; and pharmacy) and automatically generated reminders for management of dyslipidaemia in patients with coronary artery disease based on National Cholesterol Education Program-III and Israeli guidelines. The patient-specific reminders were mailed to physicians and nurses at primary care clinics. The reminders indicated the patient's risk factors, lipoprotein values, and know medications and recommended lipid lowering drug treatment if appropriate. Physicians and nurses could accept or reject CCDSS recommendations. | Usual care |
| Holbrook, 2009[26, 27], Canada | 7 | Public | Tracking of diabetes monitoring in adults in primary care. | 46 / 511 | •Primary care •Community-based clinic(18/18) | Intervention involved shared access by primary care providers and patients to a Web-based, color-coded diabetes tracker which interfaced with EMRS and an automated telephone reminder system for patients. The tracker system monitored 13 diabetes risk factors, their respective targets and gave brief, prioritised advice, based on national guidelines and a literature review. | Usual care |
| Maclean, 2009[28, 29], USA | 8 | Public | Management of diabetes in primary care. | 132 / 7412 | •Primary care •Community-based clinic (64/64) | The Vermont Diabetes Information System (VDIS) is for internal or family medicine practice providers (physicians, nurse practitioners, and physician assistants) and their patients with diabetes. Providers and patients were faxed and mailed reminders, flow sheets and reports on the management of their diabetes. The system used laboratory results on haemoglobin A1C, cholesterol, creatinine and urine protein and sent reminders when testing was overdue or results were elevated and reported on general status of diabetes. | Usual care |
| Peterson, 2008[30], USA | 10 | Public | Organization of care for primary care patients with type 2 diabetes. | 238 / 7101 | •Primary care •Community-based clinic (24/1) | CCDSS was embedded in an electronic registry and provided visit reminders, patient-specific physician alerts, a monthly progress review, and proactive support of patients at risk. This was part of a multicomponent intervention directed at patients, physicians, and clinic staff to: •Target high-risk patients •Develop Registry •Set-up Administration for staff changes •Notify patients of targets & appointments; give practitioners patient-specific reminders at visit. •Identify site coordinator •Identify local physician champion •Audit & feedback monthly •Track outcomes and activity •Educate staff. | Data collection same as for intervention. Sites received baseline data on process and outcome measures and continued usual quality improvement practices. |
| Borbolla, 2007[31], Argentina | 7 | ... | Surveillance and monitoring of blood pressure in outpatients and primary care patients with chronic disease (including hypertension, diabetes, CVD, and lipid disorders). | 182 / 2315 | •Academic centre •Hospital outpatients •Primary care (.../25) | CCDSS uses information from both EHRs and Appointment Scheduling Software to detect patients without blood pressure registries (condition I) or with high blood pressure measurements (condition II) and generate reminder lists for receptionists. Receptionists sent identified patients to assistants who assessed blood pressure, weight, height, and risk factors, reminded patients to measure blood pressure weekly and follow treatment directions, and provided educational material. All data was entered in EHRs before physician appointments. | Usual care |
| Lester, 2006[32, 33], USA | 8 | Private | Management of patients at high risk for hyperlipidaemia in primary care. | 14 / 235 | •Primary care (1/1) | CCDSS identified high-risk patients with elevated LDL cholesterol levels (> 100mg/dL 6 to 24 mo before study initiation) for cholesterol management and sent a single, customised email to physicians. Via emails, users could review patient information and, with a single click, generate a statin prescription, repeat fasting lipid profile, or decline change in medical management. CCDSS recommendations were based on evidence-based guidelines. Existing EHRs were automatically updated. | Usual care |
| Cobos, 2005[34]^c^, Spain | 10 | Private | Management of patients with hypercholesterolemia in primary care. | ... / 2221 | •Primary care (42/44) | CCDSS generated recommendations for hypercholesterolemia therapy, follow-up visit frequency, and laboratory test ordering, based on patient data entered by physicians, including cardiovascular risk and low-density lipoprotein cholesterol goals. Recommendations were adapted from the European Society of Cardiology and other societies for Hypercholesterolemia Management’s (ESCHM) guidelines. Physicians could adopt or ignore the recommendations. The intervention included availability of patient education promotions such as tablecloths and refrigerator magnets. | Usual care |
| Plaza, 2005[35], Spain | 9 | Private | Management and cost-effectiveness of asthma management in primary care. | 20 / 198 | •Subspecialty clinic •Primary care (.../5) | CCDSS provided recommendations to general practitioners and pneumologists for asthma treatment based on the Global Initiative for Asthma (GINA) guidelines. GINA based intervention included information about chronic inflammatory illness, technique when using an inhaler, maximum expiratory flow (FEM), FEM self-monitoring techniques and GINA recommendations. | Usual care |
| Sequist, 2005[36], USA | 6 | Public | Management of diabetes and coronary artery disease in primary care. | 194 / 6243 | •Academic centre •Hospital outpatients •Primary care •Community-based clinic (20/20) | When clinicians opened patient charts within EMRs, the CCDSS determined whether the patient had received care in accordance with the recommended evidence-based practice guidelines for care of diabetes or coronary artery disease. Appropriate reminders were then displayed on the patient summary screen of the EMR. Physicians could also choose to have the reminders printed. All physicians received electronic reminders for overdue preventive care services. | Electronic reminders were suppressed but printing of paper reminders was an option. All physicians received electronic reminders for overdue preventive care services. |
| Tierney, 2005[37], USA | 9 | Public | Management of asthma and COPD in adults in primary care. | 266 / 706 | •Academic centre •Primary care (4/...) | Existing computer workstations were programmed to provide care suggestions to physicians and pharmacists based on evidence-based guidelines for asthma and COPD management and data in patient EMRs. Physicians received CCDSS-generated care suggestions on paper medication lists at patient visits and on computer workstations when writing orders. Pharmacists received them electronically and could choose to do nothing or discuss suggestions with patients or physicians. They received the same educational material as the control group. | Physicians and pharmacists received a printed summary of asthma and COPD management guidelines and could attend rounds about the guidelines but did not receive care suggestions. |
| Mitchell, 2004[38], Scotland | 7 | Public | Identification, treatment, and control of hypertension in elderly patients in primary care. | … / 30345 | •Primary care (52/52) | Audit only (A) practices received “rule of halves” feedback on patients 65 to 79 years of age, including numbers of patients with blood pressure recorded, receiving antihypertensives, and with additional risk factors. Audit plus Strategic (S) practices received “rule of halves” feedback plus color-coded, patient-specific list ranked according to absolute risk of death from stroke in next 10 years for patients with a risk of ≥10%. (this is not very clear in article) | Usual care (no feedback) |
| Eccles, 2002[39, 40], UK | 10 | Public, Private | Management of asthma and angina in adults in primary care. | ... / 4506 | •Primary care (62/...) | CCDSS provided internally-developed evidence-based guidelines and care suggestions to general practitioners and practice nurses for management of adults with asthma or angina in primary care, based on electronic patient records. CCDSS was triggered when EMRs of eligible patients were opened or a relevant morbidity code was entered. | Physicians receiving asthma guidelines did not receive angina guidelines and vice versa. |
| Demakis, 2000[41], USA | 7 | Public | Screening, monitoring, and counselling in accordance with predefined standards of care in ambulatory care. | 275 / 12989 | •Other •Academic centre •Hospital outpatients (12/12) | Residents received CCDSS-generated reminders relating to 13 prespecified standards of care in 2 ways. 1) On entering a patient name into a computer terminal in the examining room, applicable reminders were automatically displayed in bold letters. 2) Applicable reminders were printed on the standard patient health summary which is attached to patient charts at visits. | Control residents only received the standard health summaries without the reminders. |
| Hetlevik, 1999[42-44], Norway | 8 | Public | Diagnosis and management for hypertension, diabetes mellitus, and hypercholesterolemia in primary care. | 56 / 3273 | •Primary care (56/...) | CCDSS provided guidance for diagnosis, history taking, physical exams, tests, and treatment based on Norwegian clinical guidelines for patients with hypertension, diabetes, or hypercholesterolemia in primary care. The CCDSS was external to, but accessible from, the main computerized medical record system and was initiated by the physician at their discretion. | Usual care |
| Lobach, 1997[45], USA | 6 | Public | Primary care of diabetes mellitus for outpatients, including screening, vaccination, and monitoring of haemoglobin A1c. | 58 / 497 | •Academic centre •Primary care (1/1) | Rule-based CCDSS used routinely collected data from individual patient EMRs to generate 8 personalised care recommendations for diabetes mellitus based on established guidelines. The recommendations were printed on ‘encounter forms’ used by clinicians to record consultation results. The program was invoked upon request for an encounter form. | Usual care |
| Mazzuca, 1990[46], USA | 7 | Public | Management of non-insulin dependent diabetes mellitus in outpatients. | 114 / 279 | •Academic centre (4/4) | 3 treatment groups: CCDSS patient-specific reminders + seminar (B); B + seminar-related clinical materials (C); and C + diabetes patient education service (D). CCDSS reminders were generated from the medical record system and placed in patients' clinic records whenever the computer detected history, physical, laboratory, or pharmacy data indicating that a seminar recommendation should be considered. | A 3.5-hour seminar covering blood sugar regulation in non-insulin dependent diabetes mellitus was offered to all physicians. All participants received a course syllabus, key reprints, and a reference book. |
| Rogers, 1984[47-49], USA | 4 | Public | Management of hypertension, obesity and renal disease in outpatients. | ... / 484 | •Academic centre •Subspecialty clinic (1/1) | CCDSS summarised patient demographics, status, and health records and made suggestions based on deficiencies in patient’s care. The 8-page patient medical summary (Northwestern University Computerised Medical Record Summary System, NUCRSS) was available to the physician at each visit. | Usual care |
| **Treatment Monitoring** | | | | | | | |
| Lo, 2009[50], USA | 10 | Public | Alerts for laboratory tests when prescribing new medications in primary care. | 366 / 2765 | •Primary care •Community-based clinic (22/1) | CCDSS generated a non-interruptive alert for missing baseline lab test when physicians ordered new medications on-line. Alerts displayed an on-screen warning in a reserved area of the screen. Providers did not have to act upon or acknowledge notifications to complete medication requests. | Usual care |
| Matheny, 2008[51], USA | 8 | Public | Routine medication laboratory monitoring in primary care. | 303 / 1922 | •Academic centre •Hospital outpatients •Primary care •Community-based clinic (20/20) | CCDSS-generated reminders for laboratory testing (potassium, creatinine, liver or thyroid function, and therapeutic drug levels) appeared on EHRs during visits of patients who were on an included medication for ≥ 365 days with no relevant laboratory test in the past 365 days. | Usual care |
| Feldstein, 2006a[52, 53], USA | 10 | Public, Private | Laboratory monitoring at initiation of specific drug treatment (ACE/ARB, allopurinol, carbamazepine, diuretic, metformin, phenytoin, pioglitazone, potassium, statins serum, or terbinafine) in primary care. | 200 / 961 | •Primary care (15/15) | 3 intervention groups: EMR, automated voice message (AVM), and pharmacy team outreach (PTO). CCDSS initiated specific baseline laboratory monitoring reminders for patients with new prescriptions for any of 10 study medications or medication classes. Reminders were delivered at baseline and 9 to 10 days later for nonrespondents. EMR reminders were sent electronically to practitioners from the chair of patient safety committee. AVM reminders were delivered via recorded telephone messages to patients, prompting them to have preordered tests completed. PTO group reminders were delivered to patients by telephone from pharmacy nurses who indicated preordered tests could be completed at designated labs. | Usual care |
| Palen, 2006[54], USA | 9 | Public | Reminders for laboratory monitoring based on medication orders in primary care. | 207 / 26586 | •Primary care (16/15) | CCDSS was integrated with EMR and CPOE systems and generated nonintrusive alert messages recommending baseline and ongoing laboratory monitoring when physicians entered orders for selected medications. | Usual care |
| Cobos, 2005[34]^c^, Spain | 10 | Private | Management of patients with hypercholesterolemia in primary care. | ... / 2221 | •Primary care (42/44) | CCDSS generated recommendations for hypercholesterolemia therapy, follow-up visit frequency, and laboratory test ordering, based on patient data entered by physicians, including cardiovascular risk and low-density lipoprotein cholesterol goals. Recommendations were adapted from the European Society of Cardiology and other societies for Hypercholesterolemia Management’s (ESCHM) guidelines. Physicians could adopt or ignore the recommendations. The intervention included availability of patient education promotions such as tablecloths and refrigerator magnets. | Usual care |
| Raebel, 2005[55], USA | 8 | Public, Private | Laboratory monitoring for initiating treatments with targeted medications in adult outpatients. | ... / 400000 | •Other (.../...) | CCDSS automatically alerted pharmacists at a call centre when targeted medications were ordered for patients who had not completed all pre-determined laboratory tests. Pharmacists reminded patients to obtain laboratory test(s) if previously ordered by physicians or ordered tests accordingly. Pharmacists notified prescribing clinicians of abnormal lab results in writing or by telephone (if urgent). | Usual care |
| McDonald, 1980[56], USA | 5 | Public | Detection of clinical events that may need follow-up (e.g., ordering a test, changing a treatment) in outpatients. | 31 / ... | •Academic centre •Hospital outpatients (1/1) | Computerized medical record system used patient data and 410 physician-developed rules, mostly related to use and follow-up of medications, to produce reports for physicians at patient visits. Reports included patient medical history and management reminders for physicians, with (R1) or without (R2) literature references. | Computer produced reminders but these were not provided to physicians. |
| McDonald, 1976[57], USA | 2 | ... | Use of laboratory tests to detect potential medication-related events in adults attending a diabetes clinic. | ... / 226 | •Academic centre •Subspecialty clinic (1/1) | CCDSS generated protocol-driven recommendations for repeat laboratory tests and treatment changes based on EMR data, including past lab results, medications prescribed, and time since previous tests. Recommendations were printed as part of patient reports and placed at the front of patient charts before visits. | Usual care |
| **Diagnosis** | | | | | | | |
| Sundaram, 2009[58], USA | 7 | Public | Risk assessment and screening for HIV in primary care. | 32 / 26042 | •Primary care (5/5) | EMR-embedded CCDSS used patient data to generate reminders for HIV risk assessments and HIV testing. Physicians and registered nurse practitioners received electronic reminders to assess HIV risk or test for HIV when they were in the patient medical record system or paper reminders on laboratory result and medication print outs. The reminders included a link to the CDC guideline for HIV testing and counseling. Electronic reminders appeared each time a patient’s medical record was opened until the practitioner completed an interactive dialog box. Providers also received electronic and paper feedback on their actions to resolve reminders every two months. All providers received an educational session on the importance of HIV screening and watched a demonstration of the CCDSS reminders. | Usual care. All providers received an educational session on the importance of HIV screening and given a demonstration of the computer-based clinical reminders. |
| Roukema, 2008[59], The Netherlands | 6 | Public | Diagnostic management for children with fever without apparent source in ED. | 15 / 164 | •Emergency Department (1/1) | CCDSS used prediction rules to generate a serious bacterial infection risk score for children < 17 years presenting to the ED with a fever without apparent source. For patients with high-risk: Users of CCDSS were given advice to “order laboratory tests” for patients randomized to CCDSS intervention. | Same as intervention except that users of CCDSS were not given “order laboratory tests” instruction for children in control group. (This is not explicitly stated in article.) |
| Downs, 2006[60], UK | 9 | ... | Investigation and management of dementia in primary care. | ... / 450 | •Primary care •Solo practice (35/35) | CCDSS was built into the EMR software for real-time, real case learning. It produced prompts for the investigation and management of dementia. (group 1). | 1. Electronic tutorial on CD Rom (self directed learning)(group 2); 2. Practice based workshops with a standard curriculum designed by a multidisciplinary expert group (peer reflection about real cases). (group 3); 3. control (usual care) (group 4) |
| Feldstein, 2006b[61], USA | 8 | Public | Guideline-recommended osteoporosis care for 50-89 year old women in primary care who experience a fracture. | 159 / 311 | •Primary care (15/1) | Patient-specific advice, based on guidelines for osteoporosis management (ordering a BMD measurement and prescribing osteoporosis medication), was delivered via EMR to primary care physicians. Providers who had not ordered a BMD measurement or medication within 3 months of first reminder received a second reminder. In 1 of 2 intervention arms, patients also received a mailed reminder with educational materials. | Usual care |
| Flottorp, 2002[62, 63], Norway | 9 | Public | Management of urinary tract infections (UTIs) in women and sore throat in primary care. | ... / ... | •Primary care (142/1) | CCDSS was not described in detail but provided support and reminders during consultations for management of UTIs and sore throats, based on locally-developed guidelines. Guidelines recommended that most patients did not need antibiotics or lab tests for sore throats and antibiotics could generally be used without lab tests in non-pregnant women with UTIs. Patients could be given advice by telephone (except for patients with a UTI who had no previous UTIs). CCDSS was part of a broader intervention that also provided treatment recommendations and patient and provider education material electronically and in print, increased telephone consultation fees, and credited participants with points for continuing medical education. | The group receiving the sore throat intervention served as controls for the group receiving the intervention for UTIs and vice versa. |
| McDonald, 1984[64], USA | 6 | Public | Cancer screening (stool occult blood, mammogram), counselling (weight reduction), immunization (influenza, pneumococcal) in addition to >1000 physician behaviour rules for outpatients. | 130 / 12467 | •Academic centre •Primary care (1/1) | CCDSS used 1491 physician-developed rules to review data in electronic medical record and produce reminder messages for physicians. Printed reports of reminders were attached to patient charts before visits. | Usual care |
| **Other** | | | | | | | |
| Thomas, 2006[15], Scotland (UK) | 8 | Public | Reminders about appropriateness of laboratory test orders in primary care. | 370 / ... | •Primary care (85/85) | 2 intervention groups. CCDSS identified requests for 9 targeted laboratory tests and automatically added locally-developed brief educational reminder messages to printed and electronic test result reports. The reminders were randomly combined with a quarterly feedback booklet that graphically presented practice-level data on ordering rates for the targeted laboratory tests compared with regional rates, and included educational messages beside each graph. Booklets were updated and mailed to family practitioners every 3 months. | 2 control groups. No feedback or quarterly feedback booklet only |
| Javitt, 2005[65], USA | 6 | Private | Management of patients when care deviates from recommended evidence-based practices in primary care. | ... / 39462 | • Primary care (.../...) | CCDSS scanned administrative data and used > 1000 decision rules to detect potential deviations from recommended care practices. Deviations triggered recommendations and supporting literature, which were sent to treating physicians. | Data about patients in the control group also triggered recommendations, but these were not sent to physicians. |
| Bates, 1999[66], USA | 8 | Public | Reduction of redundant clinical laboratory tests in hospital inpatients. | ... / 16586 | •Academic centre •Hospital inpatients (1/1) | CCDSS used data from an integrated hospital information system, including CPOE, to automatically generate reminders for physicians about potentially redundant laboratory tests when orders were entered. The CCDSS indicated if the test had recently been done or was pending, and provided results if available. The default response option was test cancellation; if physicians did not accept the reminder, they had to provide a reason from a menu selection. | Reminders were triggered by redundant tests but were not delivered. |
| Overhage, 1997[68], USA | 8 | Public | Identification of corollary orders to prevent errors of omission for tests and treatments in hospital inpatients on a general medicine ward. | 92 / 2181 | •Academic centre •Hospital inpatients (1/1) | A rule-based reminder CCDSS determined corollary orders for 87 target orders and displayed these on-line to physicians using the CPOE. Corollary orders could be accepted or rejected by physicians. | Physicians used CPOE but did not receive suggestions for corollary orders. |
| Tierney, 1988[67], USA | 6 | Public | Discourages ordering of unnecessary diagnostic tests in primary care. | 112 / 9496 | •Academic centre •Hospital outpatients •Primary care (1/1) | CCDSS embedded in CPOE system electronically displayed likelihood of abnormal test results for 8 outpatient tests, based on locally-developed statistical equations, EMRs, and data entered by physicians ordering tests. Physicians could cancel tests if desired. | Usual care. CCDSS produced predictions but these were not provided to physicians. |

Abbreviations: ACE, angiotensin-converting enzyme inhibitor; ARB, angiotensin receptor blocker; CCDSS, computerized clinical decision support system; COPD, chronic obstructive pulmonary disease; CPOE, computerized order entry system; EHR, electronic health record; EMR, electronic medical record; FEM, maximum expiratory flow; GINA, Global Initiative for Asthma; ICU, intensive care unit; NIDDM, non-insulin-dependent diabetes mellitus; NUCRSS, Northwestern University Computerised Medical Record Summary System.

^a^Ellipses (…) indicate item was not assessed.

^b^Based on 5 individual items (score 2 = yes, 1 = partly, and 0 = no) and a summed total score (range 0 to 10). Because this review update included only randomized, controlled trials, the total score differs from that reported in the previous version of this review[19]: the item evaluating study type (randomized, quasi-randomized, or concurrent controls) has been replaced by one that evaluates use of concealed allocation (concealed, unclear, not concealed).

^c^Diabetes clinic is an example of a subspecialty clinic

^d^Gives suggestions for monitoring of disease and treatment and is included in both categories. Outcomes were analyzed separately in each category but overall analysis of effectiveness (reported in text) was assessed for all diagnostic testing outcomes.
