# Supplementary material for: Can computerized clinical decision support systems improve practitioners' diagnostic test ordering behavior? A decision-maker-researcher partnership systematic review
Source: Implement Sci. 2011 Aug 3;6:88. doi: 10.1186/1748-5908-6-88 (PMC3174115; doi:10.1186/1748-5908-6-88)
Supplement: Additional file 4 — Results for CCDSS trials of diagnostic test ordering. Details results of the included studies. [file 1748-5908-6-88-S4.DOCX]

**Additional file 4, Table S4. Results for CCDSS trials of diagnostic test ordering^a^**

| **Study** | **Diagnostic Process (DP) outcomes** | **CCDSS vs control data** | **CCDSS Effect^b^ for DP** |
| --- | --- | --- | --- |
| **Disease Monitoring** | | | |
| Gilutz, 2009[25] | **Mean 21-month follow-up  1. Rate of adequate lipoprotein monitoring: % (not clear if represents patients); OR (unclear if lower & upper ranges represent 95% CIs) (primary).**  2. Effect of intervention on monitoring in 3425 patients not rehospitalized, RR, CI (not clear if 95% CI) (not prespecified). | 1. 54.8% vs 48.7%, *P*<.001; 1.28 (lower 1.17, upper 1.41), *P* < .001  2. 1.423 (1.24 to 1.64), *P*<.001 in favour of CCDSS | + |
| Holbrook, 2009[26, 27] | Median follow-up, 5.9 months  **1. Components of 8-item process composite score (out of max 10, higher scores better). (composite = primary); mean (SD) before/after intervention; mean difference (95%CI).**  Each individual component of the primary is reported in the same way (range -2 to +2) and only 6 of the 8 components are relevant for diagnostic test ordering.  **1a. Glycated Hb, measured semiannually. 1b. BP, measured quarterly. 1c. LDL-C, measured semiannually 1d. Albuminuria, measured semiannually. 1e. BMI, measured quarterly. 1f. Foot surveillance, measured semiannually.**  2. ABC (Hb, BP, and LDL-C) composite (secondary); mean (SD) before/after intervention; mean difference (95%CI).  Not prespecified  3. Patients with improvement for total process composite score; n/N, %, mean % difference. | 1a. 0.60 (0.49)/0.88 (0.33) vs 0.62 (0.49)/0.70 (0.46); 0.19 (0.09 to 0.29)  1b. 1.03 (0.79)/1.52 (0.68) vs 1.12 (0.77)/1.27 (0.74); 0.34 (0.19 to 0.49)  1c. 0.49 (0.50)/0.78 (0.42) vs 0.45 (0.50)/0.56 (0.50); 0.18 (0.07 to 0.28)  1d. 0.29 (0.46)/0.70 (0.46) vs 0.30 (0.46)/0.43 (0.50); 0.27 (0.16 to 0.39)  1e. 0.49 (0.64)/0.75 (0.75) vs 0.45 (0.64)/0.54 (0.69); 0.17 (0.02 to 0.32)  1f. 0.28 (0.45)/0.51 (0.50) vs 0.28 (0.45)/0.36 (0.48); 0.16 (0.06 to 0.25)  2. 1.80 (1.10)/2.55 (0.83) vs 1.82 (1.08)/2.08 (1.06); 0.49 (0.27 to 0.70)  3. 156/253, 61.7% vs 110/258, 42.6%; 19.1%, *P*<.001 | + |
| Maclean, 2009[28, 29] | **Mean of 32 months follow-up: 1. Proportion of tests that were timely according to guidelines (%); adjusted OR* (95% CI), (secondary). 1a. A1C (testing within 6 months if A1C<7% and 3 months otherwise).  1b. Lipids (yearly if LDL<100 mg/dl; 6 months if LDL 100-129 mg/dl; and 3 months otherwise). 1c. Serum creatinine (yearly). 1d. Urine microalbumin (yearly unless previous testing was abnormal).**  *Adjusted for baseline patient value, baseline practice performance, and clustering within practices. | 1a. 56% vs 55%; 1.17 (0.80 to 1.72), *P*=.43  1b. 74% vs 71%; 1.39 (1.08 to 1.80), *P*=.01  1c. 84% vs 80%; 1.40 (1.06 to 1.84), *P*=.02  1d. 40% vs 32%; 1.74 (1.13 to 2.69), *P*=.01 | + |
| Peterson, 2008[30] | **Prespecified**  1. Mean (SEM) change in proportion of patients having foot exams over 12 months.  2. Mean (SEM) change in proportion of patients having eye exams over 12 months.  3. Mean (SEM) change in proportion of patients having renal testing over 12 months. 4. Mean (SEM) change in proportion of patients having BP monitoring over 12 months.  5. Mean (SEM) change in proportion of patients having HbA1c testing over 12 months.  6. Mean (SEM) change in proportion of patients having LDL-C testing over 12 months.  **7. Mean (SEM) improvement in process of care index (PCI) at 12 months.**  PCI = annual BP monitoring; eye and foot exams; renal, HbA1c, and LDL-C testing. | 1. 29.4% (5.6) vs -5.6% (5.4), *P*<.001  2. 27% (2.9) vs 1.2% (2.3), *P*<.001  3. 23.2% (5.0) vs -5.3% (4.6), *P*<.001  4. 1.3% (0.9) vs -2.1% (1.4), *P*=.05  5. 2.8% (0.9) vs -5.3% (1.2), *P*<.001  6. 8.9% (1.3) vs 0.3% (1.6), *P*<.001  7. 1.29 (0.042) vs 0.22 (0.038), *P*<.001 | + |
| Borbolla, 2007[31] | **Primary outcome 1. Proportion of patients (without BP registries) with at least one BP measurement during the three months period, n (%, 95% CI).**  **2. Proportion of patients (with high BP measurements) with at least one BP measurement during the three months period, n (%, 95% CI).** | 1. 207(49.9%, 45 to 55) vs 195 (37%, 33 to 41), *P*<.001  2. 224 (61%, CI NR) vs 239 (50%, CI NR), *P*=.002 | + |
| Lester, 2006[32, 33] | **1. median (IQR) time to first measured LDL after study initiation (not prespecified)** | 1. 99 (48 to 171) vs 121 (45 to 208), *P*=.48 | 0 |
| Cobos^c^, 2005[34] | Mean follow-up 12.2 vs 11.2 months  All secondary  **1. Mean number of assessments.**  **1a. Lipid assessments.** | 1a. 1.83 vs 1.87, *P*=.30 | 0 |
| Plaza, 2005[23] | **Prespecified; 12-mo follow-up.**  **Use of the following health resources: 1. Spirometry 2. Conventional blood tests 3. Total immunoglobulin E 4. Skin allergy tests 5. Thorax radiography** | 1. 79 vs 70, *P*>.10  2. 30 vs 18, *P*>.10  3. 21 vs 2, *P*=.10  4. 17 vs 7, *P*>.10  5. 23 vs 15, *P*>.10 | 0 |
| Sequist, 2005[36] | **1. Receipt of recommended components of diabetes care during the 6 mo study, HR (95% CI) (components of primary): 1a. Annual cholesterol exam.  1b. Biennial HbAlc exam  1c. Annual dilated eye exam**   **2. Receipt of recommended components of coronary artery disease care during the 6 month study, HR (95% CI), p-value (components of primary): 2a. Annual cholesterol exam**  Note: HR>1 = benefit for CCDSS | 1a. 1.41 (1.15 to 1.72), *P*=.001  1b. 1.14 (0.89 to 1.46), *P*=.29  1c. 1.38 (0.81 to 2.32), *P*=.23  2a. 0.99 (0.75 to 1.29), *P*=.92 | 0 |
| Tierney, 2005[37] | Physician intervention vs pharmacist intervention vs both interventions vs control: Number of patients/grp, 194 vs 161 vs 182 vs 169  Primary outcome **1. Number of suggestions adhered to/Number of patients with suggestions, %, of care suggestions adhered to over 3 years. a. Obtain pulmonary function test.**  . | 1a. 6/97, 6% vs 4/65, 6% vs 9/75, 12% vs 4/66, 6%, NS | 0 |
| Mitchell, 2004[38] | No specific outcomes prespecified All outcomes: (A) pre/post vs (S) pre/post vs (C) pre/post unless otherwise stated. **1. Mean percentage of patients identified with BP identified. 1a. no record of BP 2. Among known hypertensives, mean percentage  2a. no record of BP** | 1a. 34.2%/26.3% vs 18.8%/14.2% vs 22.4%/17.9% Between group differences not significant.  2a. 19.6%/14.0% vs 3.9%/3.4% vs 10.4%/7.7% Between group differences not significant. | 0 |
| Eccles, 2002[39, 40] | **Prespecified**  **1. Adherence to angina guideline recommendations for all patients (n=2335; n=1117 computerized system, n=1218 controls) proportion of patients 12 months before/12 months after intervention period; OR (95%CI). 1a. BP recorded.  1b. Weight recorded or advised.  1c. 12 lead electrocardiogram recorded. 1d. Exercise electrocardiogram recorded. 1e. Hb concentration recorded. 1f. Thyroid function recorded. 1g. Cholesterol or other lipid concentrations recorded. 1h. Blood glucose or HbA1c concentrations recorded.**  2. Adherence to angina guideline recommendations for patients consulting during the intervention period (n=2276; n=1084 computerized system, n=1192 controls) proportion of patients 12 months before/12 months after intervention period; OR (95%CI). 2a. BP recorded.  2b. Weight recorded or advised.  2c. 12 lead electrocardiogram recorded. 2d. Exercise electrocardiogram recorded. 2e. Hb concentration recorded. 2f. Thyroid function recorded. 2g. Cholesterol or other lipid concentrations recorded. 2h. Blood glucose or HbA1c concentrations recorded.  **3. Adherence to asthma guideline recommendations for all patients (n=2363; n=1200 computerized system, n=1163 controls); proportion of patients 12 months before/12 months after intervention period; OR (95%CI)). 3a. Lung function assessed.**  4. Adherence to asthma guideline recommendations for patients consulting during the intervention period (n=2230; n=1129 computerized system, n=1101 controls); proportion of patients 12 months before/12 months after intervention period; OR (95%CI)). 4a. Lung function assessed. | 1a. 77%/80% vs 77%/80%; 1.01 (0.74 to 1.39)  1b. 23%/26% vs 24%/30%; 0.86 (0.54 to 1.35)  1c. 15%/14% vs 16%/14%; 1.01 (0.68 to 1.52)  1d. 4%/3% vs 4%/3%; 1.01 (0.56 to 1.80)  1e. 29%/33% vs 29%/33%; 1.01 (0.72 to 1.42)  1f. 17%/19% vs 18%/22%; 0.83 (0.62 to 1.12)  1g. 35%/43% vs 35%/47%; 0.85 (0.65 to 1.12)  1h. 20%/27% vs 22%/27%; 0.96 (0.67 to 1.39)  2a. 79%/82% vs 79%/82%; 1.95 (0.75 to 1.46)  2b.23%/26% vs 24% vs 30%; 0.87 (0.55 to 1.37)  2c. Only post-intervention data: 9% vs 8%; 0.94 (0.58 to 1.53)  2d. Only post-intervention data; 2% vs 2%; 1.05 (0.56 to 1.98)  2e. Only post-intervention data: 29% vs 26%; 1.08 (0.74 to 1.56)  2f. Only post-intervention data: 16% vs 16%; 0.94 (0.67 to 1.33)  2g. Only post-intervention data: 45% vs 48%; 0.87 (0.66 to 1.14)  2h. Only post-intervention data: 28% vs 28%; 0.97 (0.67 to 1.41)  3a. 43%/43% vs 42%/45%; 0.94 (0.67 to 1.33)  4a. 45%/45% vs 45%/47%; 0.94 (0.66 to 1.34) | 0 |
| Demakis, 2000[41] | **Primary outcomes 1. Proportion of patients in compliance with all 13 standards of care over 17 months. N, % adherent; OR (95% CI). 1a. Coronary artery disease, lipid levels. 1b. Hypertension: weight, exercise, sodium. 1c. Diabetes: glycosylated Hb level. 1d. Diabetes: urinalysis. 1e. Diabetes: eye exam. 1f. Diabetes of peripheral vascular disease: foot exam.  2. Proportion of all visits for which care was indicated and residents provided proper care over 17 months. N, % adherent; OR (95% CI). 2a. Coronary artery disease: lipid levels. 2b. Hypertension: weight, exercise, sodium. 2c. Diabetes: glycosylated Hb level. 2d. Diabetes: urinalysis. 2e. Diabetes: eye exam. 2f. Diabetes or peripheral vascular disease: foot exam.** | 1a. 1813, 79.0% vs 1894, 78.3%; 1.05 (0.82 to 1.34, *P*=.72) 1b. 4244, 55.2% vs 4471, 49.3%; 1.27 (0.92 to 1.75, *P*=.14) 1c.1904, 70.6% vs 2089, 65.9%; 1.24 (0.89 to 1.73, *P*=.19) 1d. 1614, 69.8% vs 1804, 62.6%; 1.38 (1.13 to 1.68, *P*=.001) 1e. 1760, 73.5% vs 1942, 63.4%; 1.60 (1.29 to 2.00, *P*<.001) 1f. 2160, 48.6% vs 2330, 42.8%; 1.26 (1.02 to 1.56, *P*=.03)   2a.833, 30.4% vs 815, 24.4%; 1.35 (1.07 to 1.71, *P*=.01) 2b. 3540, 17.0% vs 3896, 10.3%; 1.77 (1.53 to 2.05, *P*<.001) 2c. 1037, 26.5% vs 1184, 20.1%; 1.43 (1.17 to 1.77, *P*=.001) 2d. 972, 20.3% vs 1190, 16.0%; 1.34 (1.06 to 1.68, *P*=.01) 2e. 796, 17.7% vs 1094, 9.0%; 2.19 (1.63 to 2.94, *P*<.001) 2f. 2169, 13.1% vs 2201, 5.5%; 2.57 (2.02 to 3.26, *P*<.001) | + |
| Hetlevik, 1999[42-44] | Prespecified  **1. Proportion of hypertension patients (total N = 2239) without recorded data, difference (95% CI). 1a. BP over last 12 mo.  1b. Serum cholesterol over last 12 mo.  1c. BMI over 18 mo.**  **2. Proportion of diabetic patients (total N = 1034) without recorded data, difference (95% CI). 2a. BP over last 12 mo.  2b. Serum cholesterol over last 12 mo.  2c. BMI over 18 mo.  2d. HbA1c over last 12 mo.** | 1a. 14.3% vs 14.2%, 0.1 (-3.0 to 3.2)  1b. 62.3% vs 56.8%, 5.5 (1.2 to 9.8)  1c. 81.5% vs 89.2%, -7.7 (-10.8 to -4.6)  2a. 18.7% vs 18.5%, 0.2 (-5.2 to 5.6)  2b. 56.3% vs 62.7%, -6.4 (-13.2 to 0.4)  2c. 78.2% vs 93.0%, -14.8 (-19.5 to -9.9)  2d. 20.5% vs 18.8%, 1.7 (-3.8 to 7.2) | 0 |
| Lobach, 1997[45] | **1. Overall compliance with diabetes management recommendations (median % compliance; p-value)** (Primary, includes vaccinations not relevant to diagnostic test ordering) Individual recommendations:  **a. Foot examination  b. Complete physical examination  c. Chronic glycaemia monitoring  d. Urine protein determination  e. Cholesterol level  f. Ophthalmologic examination**   2. Median rate (%) clinician adherence to guidelines; p-value. [This outcome includes adherence with vaccination rates.] | 1. 32.0 vs 15.6 (from abstract); *P*=.01 a. 55.6 vs 30.0; *P*>.1 b. 33.3 vs 6.7; *P*=.05 c. 57.4 vs 52.8; *P*>.1 d. 73.3 vs 3.9; *P*=.01 e. 43.7 vs 13.4; *P*<.02 f. 18.8 vs 3.2; *P*>.1  2. 65 vs 40 (from fig. 4); *P*=.01 | 0 |
| Mazzuca, 1990[46] | Prespecified. 3 treatment groups (B = CCDSS reminder + seminar; C = B + seminar-related clinical materials; D = C + diabetes patient education service) vs control (A -seminar only).  **1. Adherence to 5 recommendations for type II diabetes care (11 months follow up): number of physicians/number of eligible patients; mean (SE) for B vs C vs D vs A. 1a. Lab order for glycosylated Hb. 1b. Lab order for fasting blood sugar. 1c. Initiation of home-monitored blood glucose.** | 1a. 114/1591; 0.24 (0.04) vs 0.37 (0.04) vs 0.25 (0.03) vs 0.21 (0.04); *P*<.05 overall, *P*<.05 C vs B, *P*<.05 D vs C  1b. 47/125; 0.80 (0.08) vs 0.69 (0.10) vs 0.70 (0.11) vs 0.68 (0.10), *P*=NS overall  1c. 114/1454; 0.11 (0.03) vs 0.16 (0.03) vs 0.14 (0.03) vs 0.06 (0.02), *P*<.05 overall but NS for individual comparisons | 0 |
| Rogers, 1984[47-49] | Prespecified  **1. Proportion of hypertension patients with medical care event at 1 year / 2 years / both years / not done. 1a. Renal function exam  1b. Potassium exam.  1c. Fundoscopic exam.  1d. Intravenous pyelogram.   2. Proportion of patient with renal disease and medical care events at 1 year / 2 years / both years / not done. 2a. Renal function exam (blood urea nitrogen, creatinine or creatinine clearance).  2b. Urine analysis.  2c. Urine culture.**   Not clearly prespecified  3. Proportion of times a diagnostic intervention result was recorded for patients with length of hospitalization available (year 1 / year 2).  3a. chest x-ray  3b. electrocardiogram  3c. urine analysis  3d. red blood cells  3e. Hb  3f. Haematocrit test; (cell pack)  3g. White blood count  3h. blood smear  3i. venereal disease research laboratory  3j. blood urea nitrogen;  3k. uric acid  3l. creatinine  3m. fasting blood sugar 3n. PCS (2 hour)  3o. cholesterol  3p. sodium  3q. potassium  3r. chlorides  3s. carbon dioxide  3t. pap smear  3u. all tests | 1a. 22.3% vs 20.5% / 9.1% vs 14.1% / 60.9% vs 50.3% / 7.6% vs 15.1%, *P*=.03  1b. 23.3% vs 20.5% / 10.2% vs 13.0% / 60.4% vs 52.5% / 6.1% vs 14.1%, *P*=.042  1c. 9.5% vs 3.2% / 59.8% vs 52.6% / 7.0% vs 4.7%/ 27.9% vs 37.8%, *P*>.05  1d. 6.5% vs 6.8% / 22.6% vs 31.6% / 39.2% vs 31.1% / 31.0% vs 28.6%, *P*>.05  2a. 18.8% vs 13.3% / 3.1% vs 13.3% / 70.3% vs 55.6% / 7.8% vs 17.8%, *P*>.05  2b. 32.8% vs 20.0% / 10.9% vs 20.0% / 46.9% vs 31.1% / 9.4% vs 28.9%, *P*<.02  2c. 48.4% vs 60.0% / 9.4% vs 11.1% / 25.0% vs 20.0% / 17.2% vs 8.9%, *P*>.05  Note: Data inconsistency.  Text (p.67, 1982 paper) indicates urine analysis significant and urine culture not significant. Table 3 states the opposite. Data were checked and the text appears to be correct.  3a. 82.1% / 84.6% vs 54.3% / 55.6%  3b. 84.6% / 80.8% vs 57.1% / 63.0%  3c. 81.6% / 80.8% vs 48.6% / 66.7%  3d. 71.8% / 73.1% vs 42.9% / 59.3%  3e. 82.1% / 73.1% vs 51.4% / 70.4%  3f. 82.1% / 73.1% vs 57.1% / 66.7%  3g. 87.2% / 73.1% vs 51.4% / 70.4%  3h. 69.2% / --- vs 28.6% / ---  3i. 25.6% / 38.5% vs 20.0% / 22.2%  3j. 87.2% / 88.5% vs 57.1% / 77.8%  3k. 84.6% / 84.6% vs 37.1% / 63.0%  3l. 87.2% / 84.6% vs 42.9% / 63.0%  3m. 84.6% / 88.5% vs 42.9% / 81.5%  3n. 18.4% / 23.1% vs 08.6% / 18.5%  3o. 87.2% / 84.6% vs 45.7% / 63.0%  3p. 82.1% / 88.5% vs 54.3% / 74.1%  3q. 82.1% / 88.5% vs 65.7% / 77.8%  3r. 82.1% / 88.5% vs 54.3% / 74.1%  3s. 82.1% / 88.5% vs 51.4% / 74.1%  3t. 61.9% / 62.5% vs 40.0% / 22.7%  3u. 75.3% / 76.2% vs 45.6% / 61.3% *P*=NR | 0 |
| **Treatment Monitoring** | | | |
| Lo, 2009 [50] | **Primary outcome.**  **1. Rate of ordering appropriate baseline laboratory tests within 14 days of clinical encounter, n/N, %; OR, 95% CI**  Not prespecified  2. Association between non-interruptive alerts and number of lab tests ordered within 14 days of alert for 11 (of 23) medication classes with >32 orders placed); n/N for both groups combined; OR, 95% CI.  2a. Antimanic agents.  2b. Hydroxymethylglutaryl-CoA;reductase inhibitors.  2c. Diuretics.  2d. ACE-inhibitor  2e. Hypoglycaemics.  2f. Antifungal antibiotics,  2g. Anticonvulsants.  2h. Antiarthritics.  2i. Cardiotonic agents.  2j, Antituberculosis agents.  2k.Angiotensin II receptor antagonists.  Not prespecified  3. Association between non-interruptive alerts and number of lab tests ordered within 14 days of alert for 5 of 12 lab tests with sufficient sample size; n/N for both groups combined; OR, 95% CI.  3a. Alkaline phosphatase.  2b. Alanine aminotransferase  3c. Thyroid stimulating hormone.  3d. Creatinine.  3e. Potassium.  Not prespecified  4. Association between non-interruptive alerts and number of lab tests ordered within 14 days of alert for 3 medications with significant associations (of 70 medications monitored); 95% CI for OR.  4a. Pravastatin.  4b. Atorvastatin.  4c. Lithium. | 1. 689/1685, 41% vs 771/1988, 39%; 1.048, 0.753 to 1.457, *P*=.78  2a. 24/71; 0.117, 0.016 to 0.858, *P*=.04  2b. 295/1025; 0.654, 0.377 to 1.136, *P*=.13  2c. 404/799; 1.324, 0.866 to 2.023, *P*=.20  2d. 289/621; 1.184 , 0.660 to 2.124, *P*=.57  2e. 82/177; 1.221, 0.662 to 2.252, *P*=.52  2f. 65/106; 0.854, 0.275 to 2.649, *P*=.79  2g. 44/255; 0.591, 0.127 to 2.756, *P*=.50  2h. 25/103; 1.328, 0.564 to 3.129, *P*=.52  2i. 35/56; 0.346, 0.024 to 4.977, *P*=.44  2j. 62/115; 1.964, 0.506 to 7.617; *P*=.33  2k. 53/130; 2.583, 0.821 to 8.131, *P*=.11  3a. 18/82; 0.740, 0.223 to 2.456, *P*=.62  3b. 483/1453; 0.789, 0.502 to 1.242, *P*=.31  3c. 17/56; 0.811, 0.235 to 2.803, *P*=.74  3d. 165/384; 1.267, 0.738 to 2.175, *P*=.39  3e. 744/1526; 1.288, 0.852 to 1.947, *P*=.22  4a. –ve association, OR CI 0.015 to 0.744, *P*=.02  4b. –ve association, OR CI 0.299 to 0.952, *P*=.03  4c. –ve association, OR CI 0.016 to 0.947, *P*=.04 | 0 |
| Matheny, 2008[51] | **Primary 1. Proportion of appropriate laboratory tests within 14 days of the clinical encounter (Medication–lab reminder): number of visits with overdue tests ordered/number of visits with overdue tests, %; adjusted OR (95% CI). 1a. Non-steroidal anti-inflammatory drug-Creatinine (8487 vs 9307 visits).  1b. Angiotensin receptor blocker-Creatinine (751 vs 832 visits).  1c. Metformin-Creatinine (856 vs 781 visits)**  **1d. Potassium supplement – Potassium (579 vs 751 visits). 1e. Potassium sparing diuretic – Potassium (761 vs 875 visits). 1f. Thiazide diuretic- Potassium (1997 vs 2508 visits). 1g. ACE inhibitor – Potassium (2279 vs 2790 visits). 1h. Statin – alanine aminotransferase (9441 vs 10935 visits). 1i. Thyroxine – Thyroid-stimulating hormone (897 vs 1233 visits). 1j. Therapeutic levels of carbamazapine, cyclosporine, Phenobarbital, phenytoin, Proc-NAPA, valproate (514 vs 755 visits).** | 1a. 150/442, 33.9% vs 136/428, 31.8%; 1.24 (0.71 to 2.15), *P*=.46  1b. 17/31, 54.8% vs 17/27, 63.0%; 0.24 (0.04 to 1.34), *P*=.10  1c. 7/20, 35.0% vs 6/16, 37.5%; 0.53 (0.05 to 5.34), *P*=.59  1d. 7/12, 58.3% vs 5/9, 55.5%; 0.91 (0.03 to 24.44), *P*=.96  1e. 13/19, 68.4% vs 17/28, 60.7%; 0.82 (0.12 to 5.60), *P*=.84  1f. 40/62, 64.5% vs 46/89, 51.7%; 1.30 (0.63 to 2.67), *P*=.47  1g. 57/119, 47.9% vs 40/80, 50.0%; 1.00 (0.43 to 2.30), *P*=.99  1h. 291/613, 47.5% vs 358/674, 53.1%; 0.89 (0.43 to 1.81), *P*=.74  1i. 22/38, 57.9% vs 25/44, 56.8%; 1.19 (0.40 to 3.53), *P*=.75  1j. 2/16, 12.5% vs 4/26, 15.4%; 0.55 (0.03 to 8.94), *P*=.68 | 0 |
| Feldstein, 2006a[52, 53] | 3 CCDSS reminder groups: Electronic medical record (EMR), automated voice message (AVM), and pharmacy team outreach (PTO).   **1. Number (proportion) of patients who completed all baseline laboratory monitoring**  1a. by day 9, immediately before second reminder **1b. by day 25 (primary)** Prespecified  2. Time to completion of lab tests: HR (95% CI). 2a. EMR vs control. 2b. AVM vs control. 2c. PTO vs control.  HR >1 indicates benefit for treatment group. | EMR vs AVM vs PTO vs Control  1a. 61/196 (31.3%) vs 117/267 (43.8%) vs 184/261 (70.5%) vs 34/237 (14.3%), *P*<.001; *P*<.05 for all differences among arms.  1b. 95/196 (48.5%) vs 177/267 (66.3%) vs 214/261 (82.0%) vs 53/237 (22.4%), *P*<.001; *P*<.05 for all differences among arms.  2a. 2.5 (1.8 to 3.5), *P*<.001  2b. 4.1 (3.0 to 5.6), *P*<.001  2c. 6.7 (4.9 to 9.0), *P*<.001 | + |
| Palen, 2006[54] | **Prespecified**  **1. Rate of compliance with ordering the recommended laboratory monitoring* for patients prescribed study medications.**  **1a. Overall, n/N dispsensings (%).**  1b. ACE-inhibitors, N dispensings (% compliance).  1c. Allopurinol, N dispensings (% compliance).  1d. Carbamazepine, N dispensings (% compliance).  1e. Colchicine, N dispensings (% compliance).  1f. Digoxin, N dispensings (% compliance).  1g. Diuretic, N dispensings (% compliance).  1h. Gemfibrozil, N dispensings (% compliance).  1i. Isoniazid, N dispensings (% compliance).  1j.Losartan potassium, N dispensings (% compliance).  1k. Metformin hydrocholoride, N dispensings (% compliance).  1l. Methotrexate, N dispensings (% compliance)  1m. Niacin, N dispensings (% compliance).  1n. Phenytoin sodium, N dispensings (% compliance).  1o. Pioglitazone hydrochloride, N dispensings (% compliance).  1p. Potassium chloride, N dispensings (% compliance).  1q. Rifampin, N dispensings (% compliance)  1r. Statins, N dispensings (% compliance).  1s. Valproic acid, N dispensings (% compliance).  Subgroup analysis (not prespecified).  2. Rate of compliance with ordering the recommended laboratory monitoring for patients prescribed study medications, %.  2a. Male patients.  2b. Female patients.  *Compliance = test completed from 180 days before to 14 days after the time of the medication order. | 1a. 10,494/18556 (56.6%) vs 8957/15686 (57.1%), *P*=.31  1b. 3099 (47.0%) vs 2729 (47.5%), *P*=.68  1c. 429 (57.6%) vs 355 (61.1%), *P*=.31  1d. 153 (34.6%) vs 119 (35.3%), *P*=.91  1e. 411 (52.8%) vs 400 (46.0%), *P*=.05  1f. 242 (55.0%) vs 178 (48.9%), *P*=.22  1g. 5384 (44.0%) vs 4270 (45.6%), *P*=.11  1h. 569 (71.2%) vs 454 (62.3%), *P*=.003  1i. 33 (15.2%) vs 36 (19.4%), *P*=.64  1j. 506 (52.0%) vs 433 (52.7%), *P*=.84  1k. 1098 (67.6%) vs 940 (7.6%), *P*=.14  1l. 7 (42.9%) vs 9 (0.0%), *P*=.03  1m. 34 (67.7%) vs 36 (47.2%), *P*=.08  1n. 83 (32.5%) vs 52 (25.0%), *P*=.35  1o. 76 (92.1%) vs 63 (93.7%), *P*=.73  1p. 1623 (54.3%) vs 1291 (57.8%), *P*=.06  1q. 7 (14.3%) vs 6 (50.0%), *P*=.20  1r. 4717 (75.7%) vs 4245 (73.9%), *P*=.05  1s. 85 (36.5%) vs 70 (38.6%), *P*=.79  2a. 57.5% vs 58.5%, *P*=.18  2b. 55.7% vs 55.9%, *P*=.82 | 0 |
| Cobos^c^, 2005[34] | Mean follow-up 12.2 vs 11.2 months  All secondary  **1. Mean number of assessments.**  **1a. Aspartate amintransferase/ alanine aminotransferase measurements**  **1b. Creatine kinase determinations.** | 1a. 1.41 vs 1.31, *P*=.03 1b. 0.54 vs 0.24, *P*=.05 | + |
| Raebel, 2005[55] | **1. Percentage (95% CI) of drug dispensings with baseline laboratory monitoring (from 180 days prior to dispensing until 14 days after) (primary outcome).** 2. n/N, Percentage (95% CI) of drug dispensings with baseline laboratory monitoring (from 180 days prior to dispensing until 14 days after) for each drug; difference (comparison by drug not prespecified) 2a. Allopurinol 2b. Amiodarone 2c. Azathioprine 2d. Carbamazepine 2e. Divalproex sodium 2f. Isotretinoin 2g. Lithium 2h. Metformin 2i. Methotrexate 2j. Nefazodone hydrochloride 2k. Pioglitazone hydrochloride 2l. Statin + gemfibrozil  Note: The number of patients started on felbamate (0 vs 2) or ticlopidine (5 vs 7) during the study was low and data on lab monitoring were not presented. | 1. 79.1% (78.0 to 80.2) vs 70.2 (68.9 to 71.5), *P*<.001  2a 575/701, 82.0% (79.9 to 84.8) vs 484/692, 69.9% (66.4 to 73.3); 12.1%; *P*<.001  2b. 202/257, 78.6% (73.1 to 83.5) vs 107/208, 51.4% (44.4 to 58.4); 27.2%; *P*<.001  2c. 97/108, 89.8% (82.5 to 94.8) vs 94/112, 83.9% (75.8 to 90.2); 5.9% *P*=.20  2d. 356/499, 71.3% (67.2 to 75.3) vs 273/484, 56.4% (51.6 to 60.7); 15.9%; *P*<.001  2e. 343/517, 66.3% (62.1 to 70.4) vs 306/514, 59.5% (55.1 to 63.8); 6.8%; *P*=.02  2f. 105/117, 89.7% (82.8 to 94.6) vs 141/148, 95.3% (90.5 to 98.1); 5.6%; *P*=.83  2g. 152/285, 53.3% (47.6 to 59.2) vs 117/272, 43.0% (37.1 to 49.1); 10.3%; *P*=.02  2h. 1538/1855, 82.9% (81.1 to -84.5) vs 1333/1759, 75.8% (73.7 to 77.8); 7.1%; *P*<.001  2i. 235/259, 90.7% (86.5 to 94.0) vs 218/246, 88.6% (84.0 to 92.3); 2.1%; *P*=.43  2j. 54/93, 58.1% (47.4 to 68.2) vs 54/112, 48.2% (38.7 to 57.9); 9.9%; *P*=.16  2k. 122/131, 93.1% (87.4 to 96.8) vs 103/115, 89.6% (82.5 to 94.5); 3.5%; *P*=.32  2l. 295/326, 90.5% (86.8 to 93.4) vs 288/345, 83.5% (79.1 to 87.2); 7.0%; *P*=.01 | + |
| McDonald, 1980[56] | **Specific reminders not prespecified for analysis. All data for reminders with (R1) or without (R2) literature references vs control  1. Number of events detected / mean adherence response rate for reminders by 17 residents over 5 weeks. 1a. Record a finding.  1b. Order a test.   2. Number of events detected / Mean adherence response rate for reminders by 9 interns over 5 weeks. 2a. Record a finding.  2b. Order a test.   3. Number of events detected / mean adherence response rate for reminders over 5 weeks by nurse clinicians. 3a. Record a finding.  3b Order a test.** | 1a. 420, 23% vs 200, 13%, *P*<.02  1b. 725, 49% vs 374, 20%, *P*<.001  2a. 101, 29% vs 49, 15%, NS  2b. 226, 38% vs 108, 9%, *P*<.02  3a. 166, 36% vs 64, 31%, NS  3b. 289, 24% vs 89, 15%, NS | + |
| McDonald, 1976[57] | **1. n/N, %, of events to which provider responded by ordering the required tests to monitor drug effects over 8 months (prespecified)**  **1a. Overall.**  1b. Renal function (blood urea nitrogen or creatinine).  1c. Serum potassium.  1d. Serum uric acid.  1e. Liver function (serum glutamic oxalacetic transaminase, alkaline phosphatase, or bilirubin)  1f. Hb or haematocrit.  1g. Leukocyte count.  1h. Serum sodium. | 1a. 144/390, 36% vs 45/402, 11%, *P*<.001  1b. 76/204, 37% vs 28/220, 14%  1c. 27/73, 36% vs 7/68, 10%  1d. 22/65, 33% vs 6/67, 9%  1e. 13/34, 38% vs 2/25, 8%  1f. 2/9, 22% vs 2/12, 16%  1g. 3/4,75% vs ?/6 (NR)  1h. 1/1, 100% vs ?/4 (NR) | + |
| **Diagnosis** | | | |
| Sundaram, 2009[58] | **1. Proportion of change in HIV testing rates (primary)**   2. Number (%) of patients tested for HIV, baseline (6 mo preintervention) / follow-up (6 mo during intervention)  *risk behaviour defined in article using US Centres for Disease Control and Prevention guidelines | 1. 0.29% vs 0.52%, *P*=.75  2. 98/5484 (1.78%) / 114/6207 (1.84%), *P*=.57 vs 67/6976 (0.96%) / 106/7375 (1.44%), *P*=.3 | 0 |
| Roukema, 2008[59] | **1. Number (proportion) of patients for whom tests were ordered (for intervention group, proportion out of cases in which CCDSS advised to order lab tests)(not clearly pre-specified)** | 1. 61 (82%) vs 40 (44%) (p value not provided but reported as significant) | + |
| Downs, 2006[60] | Prespecified; 9-months follow-up  Main outcomes  Group 1 (CCDSS) vs 2 (CD-ROM [compact disk-read-only memory]) vs 3 (Workshop) vs 4 (Control)  **1. Detection of dementia in patients who are ≥ 75 years of age: n (%).**  **2. Concordance with guidelines regarding diagnosis: n, mean (SD).**  Note: Pre-intervention detection and concordance rates were also reported; however, authors indicated these were not directly comparable because pre-intervention data were collected for up to 12 years while post-intervention data was collected for 9 months. | 1. 32 (30%) vs 11 (20%) vs 21 (31%) vs 6 (11%); CCDSS vs control, *P*=.01; Workshop vs control, *P*=.01  2. n=32 vs 11 vs 21 vs 6; 3.1 (2.4) vs 3.6 (1.4) vs 3.5 (2.4) vs 3.3 (2.0), *P*=.4 overall | + |
| Feldstein, 2006b[61] | **At 6 months 1. % of participants who received BMD measurement or osteoporosis medication within 6 months of the start of the study; p-value (primary). 1a. provider reminder + patient reminder vs control 1b. provider reminder alone vs control** 1c. provider reminder + patient reminder vs provider reminder alone  2. Change in probability of BMD measurement as predicted by linear model; coefficient (represents absolute change) (95% CI); p-value 2a. Provider reminder + patient reminder vs control  2b. Provider reminder vs control  Positive value = increase in probability of event with CCDSS.  3. Change in probability of EITHER BMD measurement or osteoporosis medication prescription as predicted by linear model; coefficient (represents absolute change) (95% CI); p-value 3a. Provider reminder + patient reminder vs control  3b. Provider reminder vs control   4. % of participants who received only BMD measurement within 6 months of the start of the study (component of primary); p-value 4a. provider reminder + patient reminder vs control 4b. provider reminder alone vs control 4c. provider reminder + patient reminder vs provider reminder alone  Note: n’s for those receiving specified treatment can be calculated from article. | 1a. 43.1% vs 5.9%; *P*<.01  1b. 51.5% vs 5.9%, *P*<.01  1c. 43.1% vs 51.5%, *P*=.88  2a. 0.31 (0.21 to 0.43)  2b. 0.39 (0.28 to 0.50)    3a. 0.38 (0.26 to 0.50)  3b. 0.47 (0.35 to 0.59)  4a. 22.9% vs 0.9%, *P*<.01  4b. 23.8% vs 0.9%; *P*<.01  4c. 22.9% vs 23.8%, *P*=.43 | + |
| Flottorp, 2002[62, 63] | **Primary outcomes for sore throat (evaluated for 18 weeks before and after the intervention) 1. Use of laboratory tests: % at follow-up (n/N), % change from baseline, % difference; intracluster correlation coefficient (95%), *P* value.   Primary outcomes for urinary tract infection (evaluated for 18 weeks before and after the intervention)**  **2. Use of laboratory tests: % at follow-up (n/N), % change from baseline, % difference; intracluster correlation coefficient (95%), *P* value.**  For both conditions, recommendations were expected to reduce testing. | 1. 42.0% (2111/5031) vs. 39.7% (1246/3135), -2.6% vs. -2.2%, 0.5%; 0.207 (0.148 to 0.266), *P*=.64.  2. 49.8% (1256/2522) vs. 55.0% (1629/2961), -3.6% vs. 1.5%, 5.1%; 0.119 (0.082 to 0.156), *P*<.05  Note: Variations in rates of antibiotic use, laboratory tests and telephone consultations are also displayed in figure 2 p.4. | + |
| McDonald, 1984[64] | Not clearly prespecified **1. Residents per-patient response to reminders over 2 years.**  **1a. Occult blood.  1b. Cervical smear.  1c. Haematocrit.  1d. Chest roentgenogram.  1e. Tuberculosis skin test.  1f. Serum K.  1g. Mammography.  1h. Reticulocytes.  1i. Iron/Iron binding.  1j. Liver enzymes.**  Note: physicians with <100 reminder messages during study were excluded from analysis, and for the 15 most frequent reminders, physicians with <6 eligible patients for an action were excluded from analysis (p.132 of article).  Note: p-values reported for 15 actions on p.134 but only 14 actions listed.  **2. For less common reminders: 2a. serum amylase for abdominal pain 2b. colon roentgenograms for Hb-positive stools 2c. urine cultures for pyurea 2d. serum fluorescent treponemal antibody tests to follow-up positive venereal disease research laboratory tests 2e. median cell volumes to detect anaemia 2f. prothrombin time after Coumadin treatment 2g. T4 index to work up findings suspicious of hypo- or hyperthyroidism**  3. Response rate (group mean response to an indication for a clinical action) amongst residents 3a. Occult blood  3b. Cervical smear 3c. Chest roentgenogram 3d. Tuberculosis skin test 3e. Serum potassium 3f. Mammography | 1. No data reported; figure 2 shows higher rates in study group for all reminders. All in favour of CCDSS group.  1a. *P*<.001  1b. *P*<.001 1c. *P*<.001 1d. *P*<.001 1e. *P*<.001 1f. *P*<.001 1g. *P*<.001 1h. *P*<.001 1i. *P*<.001 1j. *P*<.001  2. No data provided, but article specifies large CCDSS effects for the less-common reminders listed.  3a. 0.55 vs 0.22 3b. 0.38 vs 0.23 3c. 0.43 vs 0.30 3d. 0.26 vs 0.03 3e. 0.84 vs 0.75 3f. 0.08 vs 0.02 | + |
| **Other** | | | |
| Thomas, 2006[15] | **1. Median {IQR} number of targeted tests requested per 10,000 patients per practice during 12 month period (primary); OR (95% CI) for reminders with or without feedback vs feedback without reminders or control*.  1a. Total.** 1b. Autoantibody screen. 1c. Carbohydrate antigen-125. 1d. Carcino-embryonic antigen. 1e. Ferritin. 1f. Follicle stimulating hormone. 1g. Helicobacter pylori serum. 1h. Immunoglobulin E. 1i. Thyroid stimulating hormone. 1j. Vitamin B12. 2. Combined intervention effect (reminder + feedback) for total targeted test requests; OR (95% CI).  OR<1 indicates intervention group better (i.e., less likely to order targeted test). | 1. Reminders vs feedback + reminders vs feedback vs control  1a. 1317 {719 to 1590} vs 1041 {362 to 1515} vs 1079 {575 to 1818} vs 1226 {726 to 2057}; 0.89 (0.83 to 0.93), *P*=.003  1b. 36 {18 to 63} vs 31 {10 to 66} vs 33 {20 to 49} vs 41 {13 to 64}; 0.96 (0.82 to 1.12), *P*=.60  1c. 12 {4 to 23} vs 11 {4 to 19} vs 11 {3 to 19} vs 16 {9 to 25}; 0.89 (0.61 to 1.30), *P*=.54  1d. 10 {3 to 25} vs 6 {2 to 19} vs 9 {2 to 15} vs 11 {4 to 33}; 0.66 (0.44 to 0.98), *P*=.04  1e. 85 {45 to 132} vs 58 {16 to 87} vs 60 {23 to 106} vs 79 {49 to 137}; 1.04 (0.81 to 1.34), *P*=.75  1f. 55 {30 to 92} vs 49 {30 to 85} vs 57 {23 to 96} vs 77 {27 to 122}; 0.96 (0.85 to 1.09), *P*=.56  1g. 76 {38 to 98} vs 63 {20 to 117} vs 66 {21 to 104} vs 56 {36 to 98}; 0.91 (0.76 to 1.09), *P*=.29  1h. 21 {13 to 25} vs 23 {7 to 38} vs 23 {10 to 36} vs 24 {9 to 34}; 0.99 (0.79 to 1.24), *P*=.91  1i. 891 {490 to 1250} vs 800 {287 to 1077} vs 802 {432 to 1359} vs 795 {552 to 1466}; 0.82 (0.83 to 0.95), *P*=.001  1j. 29 {15 to 45} vs 19 {10 to 40} vs 23 {15 to 48} vs 34 {13 to 52}; 0.81 (0.66 to 0.99), *P*=.04  2. 0.78 (0.71 to 0.85) | + |
| Javitt, 2005[65] | 1. % physician compliance with recommendations over 12 months; relative (%) difference. (primary). **1a. Diagnostic test ordering recommendations.** | 1a. Authors indicated unable to assess, partly because 1/3^rd^ of the lab tests did not generate a discrete claim. | … |
| Bates, 1999[66] | During 4 month study period:  **1. Number (%) tests performed after reminder triggered. (primary).**  2. Test performed when reminder was triggered by test; number performed/number ordered (%). 2a. Urinalysis. 2b. Chemistry-20 profile. 2c. Urine culture. 2d. Sputum culture. 2e. Stool culture. | 1. 117437 (27%) vs 257/502 (51%), *P*<.001  2a. 35/136 (26%) vs 85/185 (46%)  2b. 37/113 (33%) vs 81/143 (57%)  2c. 22/110 (20%) vs 50/91 (55%)  2d. 14/39 (36%) vs 18/28 (64%)  2e. 3/15 (20%) vs 3/14 (21%) | + |
| Overhage{{}}, 1997[68] | 6. Compliance with the following 25 most common corollary orders within 24 hours. Total number of orders; % compliance (% increase) (not prespecified).  6a. Serum creatinine  6c. Serum electrolytes  6d. Glycosylated haemoglobin A1.  6f. Serum glutamic pyruvic transaminase (alanine aminotransferase)  6h. Serum glutamic oxaloacetic transaminase (aspartate amintransferase)  6i. Capillary glucose.  6j. Blood cell profile.  6k. Stool occult blood test  6m. Theophylline level  6o. Platelet count  6q. Reticulocyte count  6s. Fe-TIBC  6u. Phenytoin level  6v. Portable anterior-posterior chest x-ray  6w. arterial-venous blood gas  6x. Simplate bleed time  6y. Gentamicin level | 6a. 1209; 48.28% vs 41.18% (7.10%)  6c. 1034; 87.03% vs 70.86% (16.18%) 6d. 821; 23.71% vs 7.39% (16.32%)  6f. 569; 12.63% vs 1.87% (10.76%)  6g. 506; 79.35% vs 79.26% (0.09%) 6h. 467; 7.14% vs 0% (7.14%) 6i. 446; 30.77% vs 4.41% (26.36%) 6j. 382; 80.46% vs 51.44% (29.02%) 6k. 374; 60.94% vs 12.09% (48.85%)  6m. 270; 75.89% vs 46.51% (29.38%)  6o. 236; 70% vs 15.09% (54.91%)  6q. 205; 19.66% vs 11.36% (8.29%)  6s. 149; 12.64% vs 0% (12.64%) 6t. 143; 90.74% vs 65.17% (25.57%)   6v. 127; 81.69% vs 33.93% (47.76%) 6w. 123; 72.60% vs 0% (72.60%) 6x. 123; 26.23% vs 0% (26.23%) 6y. 118; 90% vs 75.86% (14.14%) | … |
| Tierney, 1988[67] | **Not prespecified**  **1. Mean (SEM) probability of abnormal study test over 6 months.** | 1. 0.24 (0.006) vs 0.18 (0.005), *P*<.001 | + |

Abbreviations: ACE, angiotensin-converting enzyme; BMD, bone mineral density; BMI, body mass index; BP, blood pressure; CCDSS, computerized clinical decision support system; CI, confidence interval; Hb, haemoglobin; HIV, human immunodeficiency virus; HR, hazard ratio; IQR, interquartile range; LDL-C, low-density lipoprotein cholesterol; NR, not reported; NS, not significant; OR, odds ratio; RR, relative risk; SD, standard deviation; SE, standard error; SEM, standard error of the mean.

^a^Ellipses (…) indicate item was not assessed or could not be evaluated. Outcomes in bold font were assessed for effect.

^b^Outcomes are evaluated for effect as positive (+) or negative (−) for CCDSS, or no effect (0), based on the following hierarchy. An effect is defined as ≥ 50% of relevant outcomes showing a statistically significant difference (2*P* < .05):

- If a single “primary outcome” (defined in the methods section of the manuscript) is reported, *in which all components are applicable*, this is the only outcome evaluated.
- If > 1 primary outcome is reported, the ≥ 50% rule applies and only the primary outcomes are evaluated.
- If no primary outcomes are reported (or only some of the primary outcome components are relevant) but overall analyses are provided, the overall analyses are evaluated as primary outcomes. Subgroup analyses are not considered.
- If no primary outcomes or overall analyses are reported, or only some components of the primary outcome are relevant for the application, any reported prespecified outcomes are evaluated.
- If no clearly prespecified outcomes are reported, any available outcomes are considered.
- If statistical comparisons are not reported, ‘effect’ is designated as not evaluated (…).

^c^Gives suggestions for monitoring of disease and treatment and is included in both categories. Outcomes were analysed separately in each category but overall analysis of effectiveness (reported in text) was assessed for all diagnostic testing outcomes.
