# Supplementary material for: Can computerized clinical decision support systems improve practitioners' diagnostic test ordering behavior? A decision-maker-researcher partnership systematic review
Source: Implement Sci. 2011 Aug 3;6:88. doi: 10.1186/1748-5908-6-88 (PMC3174115; doi:10.1186/1748-5908-6-88)
Supplement: Additional file 5 — Costs and CCDSS process-related outcomes for trials of diagnostic test ordering. Cost and CCDSS process-related outcomes for the included studies. [file 1748-5908-6-88-S5.DOCX]

**Additional file 5, Table S5. Costs and CCDSS process-related outcomes for trials of diagnostic test ordering^a^**

| **Study** | **CCDSS adverse effects** | **Costs** | **Group comparison for CCDSS workflow** | **Practitioner satisfaction with CCDSS system** |
| --- | --- | --- | --- | --- |
| **Disease Monitoring** | | | | |
| Gilutz, 2009[25] | Author comment: no adverse effects. | Author comment: yes it was cost effective; data not stated. The cost of implementing the CCDSS was $170 000. | ... | Author commented that 143 satisfaction grading forms were available with 91% rate of general satisfaction. |
| Holbrook, 2009[26, 27] | ... | ... | ... | Many providers indicated that technical difficulties with the CCDSS had a negative impact on the perceived usefulness of the system. However, 16/33 (48%) felt they improved their knowledge of diabetes targets, 11/33 (33%) felt their patients adherence to appointments improved, and 12/33 (36%) felt patient access to high-quality diabetes care improved. |
| Maclean, 2009[28, 29] | ... | Estimated total costs of care per person per year: $3,202 vs $4,937; adjusted difference, -$2,426 (95% CI -$4,647 to -$205), *P*=.033. Ongoing information technology support estimated at < 1 hour/month. | ... | ... |
| Peterson, 2008[30] | ... | Costs: 1 hour/provider for coordinators, 1 hour/month for local physician champions, software for 67% of sites, computer + printer for 33% of sites. | ... | No formal measure of practitioner satisfaction; however, author reported that a majority appeared satisfied with the system. |
| Borbolla, 2007[31] | Author commented that no adverse effects were found. | ... | ... | Author commented that informal evaluations found practitioners were satisfied. |
| Lester, 2006[32, 33] | ... | Median time to complete an email notification: 90 sec (range 15 sec to 49 min). | ... | ... |
| Cobos, 2005[34]^c^, Spain | ... | Direct costs = sum of costs of physician visits, laboratory analyses, and lipid lowering drugs prescribed during the study. For each patient, visit and laboratory costs were estimated by frequency x unit cost (physician visit 12€, lipid 9.46€, alanine aminotransferase and aspartate amintransferase 2€ each, creatine kinase 1€). All costs in €s.  1. Lipid-lowering drug treatment costs at 1 year. 125,569 vs 214,683  2. Lipid lowering drug total costs at 1 year. 170,061 vs 264,658  3. Adjusted means for treatment costs per patient; difference (95% CI); savings %. 178 vs 237; 59 (34 to 83, *P*<.001); 24.9%.  4. Adjusted means for total costs per patient; difference (95% CI); savings %. 223 vs 283; 60 (33 to 86), *P*=.001; 20.8% | ... | ... |
| Plaza, 2005[35] | ... | Direct and indirect costs were calculated. The former was calculated as the product of the consumption of each resource times its unitary cost. Treatment costs were estimated using drug market prices in Spain. The remaining costs were obtained through the SOIKOS database with prices corrected for year 2001 and using average prices (medical visits: 8.47 €; home visits:19.53 €; emergency visits: 87,53 €; days in ICU: 1,156.44 €; shift days: 282.46 €; espirometries: 10.03 €; blood standard analysis: 10.73 €; total E immunoglobulin: 6.77 €, thorax radiographies: 14.35 €; skin allergic tests: 30.05 €).  Indirect costs were calculated, for active workers, as the product of hours lost and the total cost per hour of effective work (11.79 €).  The total costs were estimated using a social and national healthcare system perspective. From a social perspective the medical costs per patient rose to 2444.35 (987 treatments) in the PCH group and 1408 (833 treatments) in group 1. The estimated difference was -1022 (95%IC -2165 to 122; *P*=.08).  From the perspective of the National Health Care System, the total median medical cost per patient rose 1544 (891 treatments) in the PCH group and to 1077 (767 treatments) in group 1. The difference was -489 (95%IC -1302 to 324; *P*=.24). . | ... | ... |
| Sequist, 2005[36] | ... | ... | ... | 71% of physicians preferred the electronic decision support over a paper-based system and 76% thought that the system helped to improve quality of care. Of the physicians in the intervention group, 68% found electronic reminders for diabetes care useful; 53% found them useful for coronary artery disease management. |
| Tierney, 2005[37] | ... | Physician intervention vs pharmacist intervention vs both interventions vs control  1. Mean (SD) direct health care charges over 12 months (US $). All *P*=NS unless noted otherwise. 1a. Outpatient charges. 3,142 (3,381) vs 2,814 (3,282) vs 3,177 (3,558) vs 3,129 (2,921); 1b. Inpatient charges. 4,864 (17,257) vs 2,519 (7,267) vs 2,475 (8,699) vs 2,671 (6,805); 1c. Total health care charges. 8,006 (18,720) vs 5,333 (9,400) vs 5,652 (10,579) vs 5,800 (8,536), *P*<.05 for increase with physician intervention. | ... | ... |
| Eccles, 2002[39, 40] | ... | ... | ... | Author comment: We have always interpreted Figure 3 (shows the number of times the guidelines were triggered for each practice and the proportion of active interactions that involved going beyond the first screen - the median number of active interactions was zero for much of the study) to mean that the majority of the users were not satisfied - in that they stopped using the system. This is certainly what the process evaluation suggested. |
| Hetlevik, 1999[42-44] | ... | ... | ... | 1. Physicians reported the CCDSS provided some or much benefit for checking blood pressure (68%); taking history (67%); diagnostic support, lab tests, lifestyle advice (oral), and infarction risk score calculation (61%); lifestyle advice (out-prints) (56%); clinical exams (53%), and treatment indications (50%).  2. Physician evaluation of CCDSS user-friendliness (agreed or partly agreed).  a. One CCDSS for 3 diagnoses as a good/acceptable solution (20/24, 83%)  b. CCDSS too large (22/24, 92%)  c. Recommended procedures too time-consuming (20/24, 83%)  d. Many recommendations were unnecessary (12/24, 50%)  e. Could remember procedures without the CCDSS (16/24, 67%)  3. Physicians reported some CCDSS implementation strategies were of some or large benefit:  a. Ready to use (11/21, 52%)  b. Physician training (17/21, 81%)  c. Assistant training (10/18, 56%)  d. Physician user manual (11/21, 52%)  e. Telephone repetitions for physicians (15/21, 71%)  f. Attending risk intervention seminar (2/2, 100%)  Strategies of little of no use:  g. Assistant user manual (10/19, 53%)  h. Report of own patients with diabetes (11/21, 52%)  i. Checking use of CDSS (14/20, 70%)  j. Visiting the CCDSS stand (2/2), 100%). |
| Lobach, 1997[45] | … | … | No difference in encounter length when CCDSS was or was not supplied (*P*>.1). | … |
| **Treatment Monitoring** | | | | |
| Cobos, 2005[34]^c^, Spain | ... | Direct costs = sum of costs of physician visits, laboratory analyses, and lipid lowering drugs prescribed during the study. For each patient, visit and laboratory costs were estimated by frequency x unit cost (physician visit 12€, lipid 9.46€, alanine aminotransferase and aspartate amintransferase 2€ each, creatine kinase 1€). All costs in €s.  1. Lipid-lowering drug treatment costs at 1 year. 125,569 vs 214,683  2. Lipid lowering drug total costs at 1 year. 170,061 vs 264,658  3. Adjusted means for treatment costs per patient; difference (95% CI); savings %. 178 vs 237; 59 (34 to 83, *P*<.001); 24.9%.  4. Adjusted means for total costs per patient; difference (95% CI); savings %. 223 vs 283; 60 (33 to 86), *P*=.001; 20.8% | ... | ... |
| McDonald, 1976[57] | There was a problem with the recommendations for renal treatment, such that in some cases the computer suggestion unwarranted reductions in medication. Practitioners did not act on the erroneous recommendations. | ... | ... | ... |
| **Diagnosis** | | | | |
| Sundaram, 2009[58] | … | … | … | 78% of the intervention providers who received feedback felt it had an effect on their HIV test ordering practices. |
| Flottorp, 2002[62, 63] | Experience with installation and use of software was reported in the supplementary paper. Practices provided group feedback on the following 4 items (5-point scale, 1 = no problems, 5 = very difficult).  1. Experience with software installation (n=112): mean 2.5 (median 2); 1, 35%; 2, 18%; 3, 23%; 4, 9%; 5, 13%; don't know, 3%  2. Experience using software (n=112): mean 2.1 (median 2); 1, 39%; 2, 29%; 3, 18%; 4, 8%; 5, 4%; don't know, 3%  3. Experience filling out 'pop-up' questionnaires (n=112): mean 1.9 (median 2); 1, 42%; 2, 30%; 3, 19%; 4, 5%; 5, 2%; don't know, 2%  4. Software worked technically ok (n=120): mean 2.1 (median 2); 1, 32%; 2, 36%; 3, 21%; 4, 11%; 5, 1%; don't know, 0% | ... | ... | Limited information on practitioner satisfaction with the CCDSS in this multicomponent intervention. Mean score of 2.8 (range 1 = yes, 5 = no) for 205 respondents on question "Was the computer-based advice helpful?" |
| **Other** | | | | |
| Thomas, 2006[15] | ... | ... | ... | Author reports that approximately 95% of general practitioners liked the system. |
| Javitt, 2005[65] | ... | Study reported payer costs but not patient or caregiver costs. Intervention costs were $1 to $1.50 per plan member per month (pmpm) with an overall return on investment of $8.07 pmpm.  Other cost outcomes for N = 19,739 vs 19,723 patients. 1. Total inpatient charges per patient per month over 12 months; difference in $. $58.95 vs $68.36; -$9.41 (*P*=.001). 2. Total inpatient paid claims per patient per month over 12 months; difference in $. $26.06 vs $28.20; -$2.14 (*P*=.008). 3. Overall difference ($ per patient per month) in charges over 12 months (95% CI). -$18.62 (-$12 to -$25), p-value not reported. 4. Overall difference ($ per patient per month) in paid claims over 12 months (95% CI) (primary). -$8.07 (-$5 to -$11), p-value not reported.  Subgroup analyses of patients who triggered recommendations (both intervention [n=961] and control [n=982]): 5. Total inpatient charges per patient per month over 12 months; difference in $. $242.30 vs $296.30; -$54.00 (*P*=.007). 6. Total inpatient paid claims per patient per month over 12 months; difference in $. $93.50 vs $127.50; -$34.00 (*P*=.006). 7. Overall difference ($ per patient per month) in charges over 12 months (95% CI). -$77.91 (-$26 to -$130) (*P*=.003). 8. Overall difference ($ per patient per month) in paid claims over 12 months (95% CI). -$68.08 (-$39 to -$98) (*P*=.003). 9. Difference in charges/paid claims over 12 months by type of intervention recommended. 9a. Add a drug. Higher in control group (data shown only in figure 2, $300-350/approx $200) 9b. Stop a drug. Higher in control group (data in figure 2, $100-$150/$50-$100 9c. Get a test. NS (data in figure 2, 0-$50/0 to -$50 Note: Figures (but no data) in article show claims costs separately for inpatients, outpatients, professional, pharmacy, and total – both overall and for subgroup of patients who triggered recommendations.  10. Difference in charges/paid claims over 12 months for subgroup of patients who did not trigger care considerations. (Not significant). Subgroup analyses for patients with HOPE trial-consistent recommendation for ACE-I prescription (n=156 vs 155 patients). 11. Inpatient charges per person over 12 months, mean. $5,835 vs $8,746, *P*=.05 12. Non-HOPE-related hospital inpatient charges per person over 12 months, mean. $6,704 vs $8,416, *P*=.30 | ... | ... |
| Bates, 1999[66] | Cancellation of tests was assessed for adverse events by checking for new abnormal test results for the same test performed within 72 hours of the cancellation. 119/224 (53%) of accepted reminders were followed by the same type of test within 72 hours; 55 (24%) were abnormal although only 8 (4%) provided new information and 2 (1%) led to a change in clinical management. | (Estimated) annual charge savings $35,000 from reduction in redundant tests, where 1994 charges for each test were used for the calculation. | ... | From previous finding, CPOE satisfaction is high (mean >5 on 1 to 7 scale).  Satisfaction with reminders for redundant tests was lower (3.5 on 1 to 7 scale). |
| Overhage, 1997[68] | ... | Mean hospital charges for intervention vs control: $8,073.52 vs $8,589.47 (difference -$515.95, 95% CI -828.41 to 1,316.85, *P*=.68). | ... | ... |
| Tierney, 1988[67] | ... | 1. Mean (SEM) charge for tests per patient visit over 6 months ($ US, % change). CCDSS vs control  1a. Overall. 11.18 (0.59) vs 12.27 (0.63), -8.8%, *P*<.05 [Note 11.20 vs 12.28 in table 3]  1b. Electrolyte levels. 3.83 vs 4.27, -10.3%, *P*<.05  1c. Complete blood cell count. 1.66 vs 1.87, -11.2%, *P*<.05  1d. Chest roentgenogram. 2.63 vs 3.05, -13.8%, *P*=NS  1e. Urinalysis. 0.28 vs 0.24, 14.5%, *P*=NS  1f. Electrocardiogram. 1.19 vs 1.15, -3.8%, *P*=NS  1g. White blood cell differential count. 0.25 vs 0.26, -8.0%, *P*=NS  1h. Thyroid-stimulating hormone concentration. 1.12 vs 1.25, -10.3%, *P*=NS  1i. Urine culture. 0.24 vs 0.19, 26.8%, *P*=NS  Note: 1f. difference reported as -3.6 but looks like it should be +3.6. % differences also seem off for urinalysis through urine culture. 1st few consistent using formula: (ctrl value - intervention value) / ctrl value but then: e.g. urinalysis difference = (0.24 - 0.28) / 0.24 = +16.7 rather than 14.5. (if the intervention value was used as the denominator, it works out as 14.3% - but looks like the control data should be the denominator). Biggest difference for WBC diff for 0.26 vs 0.25 is 3.8% not 8.0%. | ... | ... |

Abbreviations: CCDSS, computerized clinical decision support system; CI, confidence interval; SD, standard deviation; CPOE, computerized order entry system; SGRQ, St. George’s Respiratory Questionnaire; SEM, standard error of the mean.

^a^Ellipses (…) indicate outcome was not assessed.

^b^Costs include workflow measures such as time to process alerts if these are not directly compared between groups.

^c^Gives suggestions for monitoring of disease and treatment and is included in both categories. Outcomes were analyzed separately in each category but overall analysis of effectiveness (reported in text) was assessed for all diagnostic testing outcomes.
